# Entomological investigations into yellow fever outbreak in northern Ghana

**DOI:** 10.1371/journal.pgph.0007346

**Published:** 2026-10-15

**Authors:** Joseph Harold Nyarko Osei, Sellase Pi-Bansa, Kwadwo Kyereme Frempong, Mavis Ofei, Helena Anokyewaa Boakye, Jane Ansah-Owusu, Sandra-Candys Adwirba Akorful, Richard Odoi-Teye Malm, Christopher Nii Laryea Tawiah-Mensah, Mufeez Abudu, Andy Asafu-Adjaye, Seth Offei Addo, Bright Agbodzi, Ronald Bentil, Deborah Pratt, Shirley Nimo-Paintsil, Joseph Humphrey Kofi Bonney, Maxwell Alexander Appawu, Millie-Cindy Aba Aude Koffi, Sylvester Coleman, Millicent Captain-Esoah, Chrysantus Kubio, Daniel Adjei Boakye, Samuel Kweku Dadzie

**Affiliations:** 1 Department of Parasitology, Noguchi Memorial Institute for Medical Research, College of Health Sciences, University of Ghana, Legon, Ghana; 2 Biomedical and Public Health Research Unit, Water Research Institute, Council for Scientific and Industrial Research, Accra, Ghana; 3 Department of Biological, Environmental and Occupational Health, School of Public Health, College of Health Sciences, University of Ghana, Legon, Ghana; 4 Department of Animal Biology and Conservation Science, College of Basic and Applied Sciences, University of Ghana, Legon, Ghana; 5 Acute Febrile Laboratory, Naval Medical Research Unit 3, Noguchi Memorial Institute for Medical Research, College of Health Sciences, University of Ghana, Legon, Ghana; 6 Department of Molecular Medicine, School of Medicine and Dentistry, Kwame Nkrumah University of Science and Technology, Kumasi, Ghana; 7 Department of Virology, Noguchi Memorial Institute for Medical Research, College of Health Sciences, University of Ghana, Legon, Ghana; 8 Department of Theoretical and Applied Biology, College of Science, Kwame Nkrumah University of Science and Technology, Kumasi, Ghana; 9 Liverpool School of Tropical Medicine, Liverpool, United Kingdom; 10 Department of Applied Biology, School of Environment and Life Sciences, University of Technology and Applied Sciences, Navrongo, Ghana; 11 Savannah Regional Health Directorate, Damongo, Ghana; PLOS: Public Library of Science, UNITED STATES OF AMERICA

## Abstract

Arboviruses are pathogens of emerging/re-emerging infectious diseases. Half the world’s population is at risk. Recent African epidemics confirmed 19,569 cases with 820 deaths. In Ghana, Savannah Region had yellow fever (YF) outbreak in 2021. Reports revealed 35 deaths, 202 suspected cases (case fatality rate (CFR) = 17%), and 70 confirmed cases (CFR = 50%). This paper presents YF outbreak entomological investigations findings. A team with expertise in YF outbreak response were assembled and tasked to assess, contain and/or control the rapid spread of YF. Immature *Aedes aegypti* were collected in/around houses, and adult sampled using BG-Sentinel traps, oral aspirators and Prokopack. The chikungunya, dengue fever, YF, and Zika viruses screening using real time reverse-transcription PCR (detection limit <10 RNA copies/reaction) followed mosquitoes morphological identification. Breeding containers (12,264) were examined. Larvae (3,885) and pupae (1,186) were obtained from 173 containers (positivity rate = 1.26%). Larvae were identified with short and stout siphons. Pupae had short but distinct serrated spicules on paddle fringe and branched/fanlike (6–7 branches) seta 9-VIII. Out of 1,001 houses surveyed, 130 were positive for larvae/pupae (positivity rate = 12.99%). Receptacles included plastic [46.95% (6,529)], metallic [43.32% (6,024)], clay jar [5.42% (753)], tire [4.06% (565)], and well [0.24% (34)]. WHO larval indices thresholds (BI, CI, HI, and PDI) were used to assess risk. Exactly 1,571 adults were identified [*Aedes aegypti aegypti* (35), *Aedes aegypti formosus* (619), and *Culex* sp. (917)]. Outdoor collection were higher (86.4%) than indoors (13.6%). No arboviruses were detected. Generally, vector indices/densities suggested high-risk arbovirus transmission although vectors were not arbovirus positive. *Aedes aegypti formosus* was dominant. They are bridge vectors maintaining sylvatic YF cycles and spillover transmission. High risk for YF poses public health threat that calls for *Aedes* mosquito surveillance systems and vector control strategies. *Ae*. *ae*. *formosus* might be drivers for outbreak and/or YF transmission.

## Introduction

Globally, approximately half the human population on earth is at risk of arboviral infections with recent epidemics in Africa documenting several cases and deaths (CFR = 4.2%) [1]. *Aedes*-borne viruses (ABVs) are arboviruses with estimated annual cases of > 77.7 million, mortality of > 42,000, and ~ 2.3 million disability life years (DALYs) [2,3]. About 17% of infectious diseases worldwide are arthropod-borne and a significant number are the emerging vector-borne diseases [4–7]. It has also been estimated that more than 1.0 billion people in Africa are at risk of arboviral diseases [8–11]. In recent times, certain countries in West Africa have experienced outbreaks of arboviruses such as dengue fever (DF) and yellow fever (YF). These viruses belong to the family *Flaviviridae*. They are enveloped RNA viruses and the commonest global human arboviruses [12]. In tropical Africa, the yellow fever virus (YFV) affects primates (people and monkeys) [13]. YFV evolved in Africa’s sylvatic cycles, where the disease is maintained between nonhuman primates and forest-dwelling *Aedes* mosquitoes, and has a history of continuous transmission among humans via *Aedes aegypti* [14]. The urban cycles on the other hand is maintained between humans and urban-dwelling *Aedes aegypti* mosquitoes. YFV can cause asymptomatic infection, non-specific clinical disease, and deadly haemorrhagic fevers in humans. For instance, the recent yellow fever outbreak in the Savannah Region of Ghana recorded a case fatality rate (CFR) of 50%. Despite the development and presence of a safe vaccine, there was an outbreak in Ghana [15]. There were pockets of unvaccinated (nomadic and mobile) populations resulting in vaccine coverage gaps below national target though within limits for herd protection [16]. There are reported incidence and epidemics of common arboviruses including yellow fever (YF) in several African countries [17–19] as well as in sub-Saharan Africa [20] and Ghana is not an exception (see S1 Table).

Ghana is surrounded by countries known to be endemic for arboviruses [18] and yellow fever. The recent yellow fever and other arbovirus outbreaks were in Cote d’Ivoire, Burkina Faso, and Togo occurred in 2016, 2016/2017, and 2020 respectively [18,21,22]. There had also been historical reports of such incidence and epidemics in these neighbouring countries closest to Ghana [23]. However, there had been no confirmed outbreak and rare cases of DF detection locally and in travellers during the years 1932, 1987–1993, and 2002–2005 [17,23]. Recently, there has been reports of YF outbreak in a couple of African countries [19,24] (see Supporting Information, S1 Table). In the year 2021, several African countries had another round of YF outbreak [25]. Ghana in 2021 also had a yellow fever outbreak which started in the Savannah Region and quickly spread to the Upper West, Bono, and Oti regions all in the northern parts of Ghana. The first case of the YF outbreak was reported on the 15^th^ of October 2021 reaching the third region by 27^th^ of November 2021. Within this period, as many as 202 suspected cases were reported. Out of this number, 70 were confirmed to be YF positives and 35 deaths were reported out of the confirmed cases [26]. The reported CFR for the suspected cases in the study districts was 17% but 50% for confirmed cases. The yellow fever outbreak was concentrated in North Gonja and West Gonja districts with epidemiological investigations repeatedly identifying people from different communities such as Busunu, Daboya, Damongo, Kagbal, and Kawankura among others [26]. These communities were found near or within forested ecological zones notably condusive for sylvatic transmission cycles between the *Aedes* mosquito vectors and non-human primates [26].

Yellow fever viruses are transmitted to people mainly by *Aedes aegypti* in tropical and subtropical areas around the globe [27]. Mosquitoes of the *Aedes* genus are vectors for several emerging diseases that are expanding geographic range [28]. Some of these mosquito species are anthropophilic and endophilic in most parts of the world [29] therefore increasing vector-human contact resulting in efficient disease transmission and they are also invasive. There are few *Aedes* mosquito species that are invasive. These include *Aedes aegypti*, *Ae*. *albopictus*, *Ae*. *japonicus*, *Ae*. *koreicus*, *Ae*. *atropalpus*, and *Ae*. *triseriatus* [30]. Out of these invasive mosquito species, *Ae*. *aegypti* is the primary vector for yellow fever while *Ae*. *albopictus* is a potential vector [31]. Changing environmental variables and urbanization has contributed immensely to the spread of these *Aedes* mosquitoes [32]. *Aedes aegypti aegypti* have been described as the domestic subspecies that have a preference for human blood, while *Aedes aegypti formosus* is known to be an efficient vector also found in sub-Saharan areas [33] and in forested areas. These characteristics make them significant vectors of arboviruses like yellow fever virus (YFV), dengue fever virus (DENV), Zika virus (ZIKV), and chikungunya virus (CHIKV). These vectors could be used as predictors for future outbreaks and hence the need to carry out surveillance [34–36]. Ghana’s response to the 2021 outbreak was right in the sense that stakeholders of health / experts from diverse fields of study were put together as quickly as possible to handle the menace of the YF outbreak. This research was to carry out the vector study to have information on the risk of infections. Measures were put in place to control the spread and mortality due to YF by the national emergency response team.

Most arboviral infections do not have vaccines and/or medications and are at best managed. Outbreaks of arboviral diseases are therefore a major public health concern globally. It is therefore imperative that we assess the risk of arboviral transmission through an established system of continuous surveillance while waiting for the development of vaccines and/or medications for arboviral diseases. Till date, supportive care has been the standard for the management of arboviral diseases. In this paper, we investigated the outbreak of YF in the Savannah Region by assessing the entomological risk after the YF outbreak to identify the *Aedes* vectors, determine vector distribution and densities, detect yellow fever virus infections in *Aedes* mosquitoes, and evaluate entomological factors and breeding sites for risk of yellow fever virus transmission. We anticipated high distribution and densities of breeding sites with *Aedes aegypti* vectors being responsible for the quick spread of yellow fever during the outbreak. This study was also to generate data that will inform the need to establish an *Aedes* mosquito surveillance system for Ghana. The data established will also inform targeted *Aedes* vector control interventions. The study findings will give valuable data to support public health systems to contain other outbreaks that may occur subsequently within the borders of Ghana and other endemic countries that might experience similar outbreaks in the future.

## Methods

### Ethics statement

This outbreak and follow-up investigation was carried out in collaboration with the Ghana Health Service and National Malaria Elimination with ethical approval from the Ghana Health Service Ethical Commitee (Approval Number:GHS-ERC:023/03/21). Verbal consent was obtained for community entry; from households heads to conduct mosquito surveys in houses with the members of the households as the respective witnesses; and from human landing catches volunteers (who were all trained entomologists and vaccinated against yellow fever virus) for mosquito collections. All activities done in the field were approved by the Institutional Review Board.

The study was conducted in 4 districts of the Savannah Region. These districts included West Gonja, Central Gonja, North Gonja, and Sawla-Tuna-Kalba districts (Fig 1).

**Fig 1:**
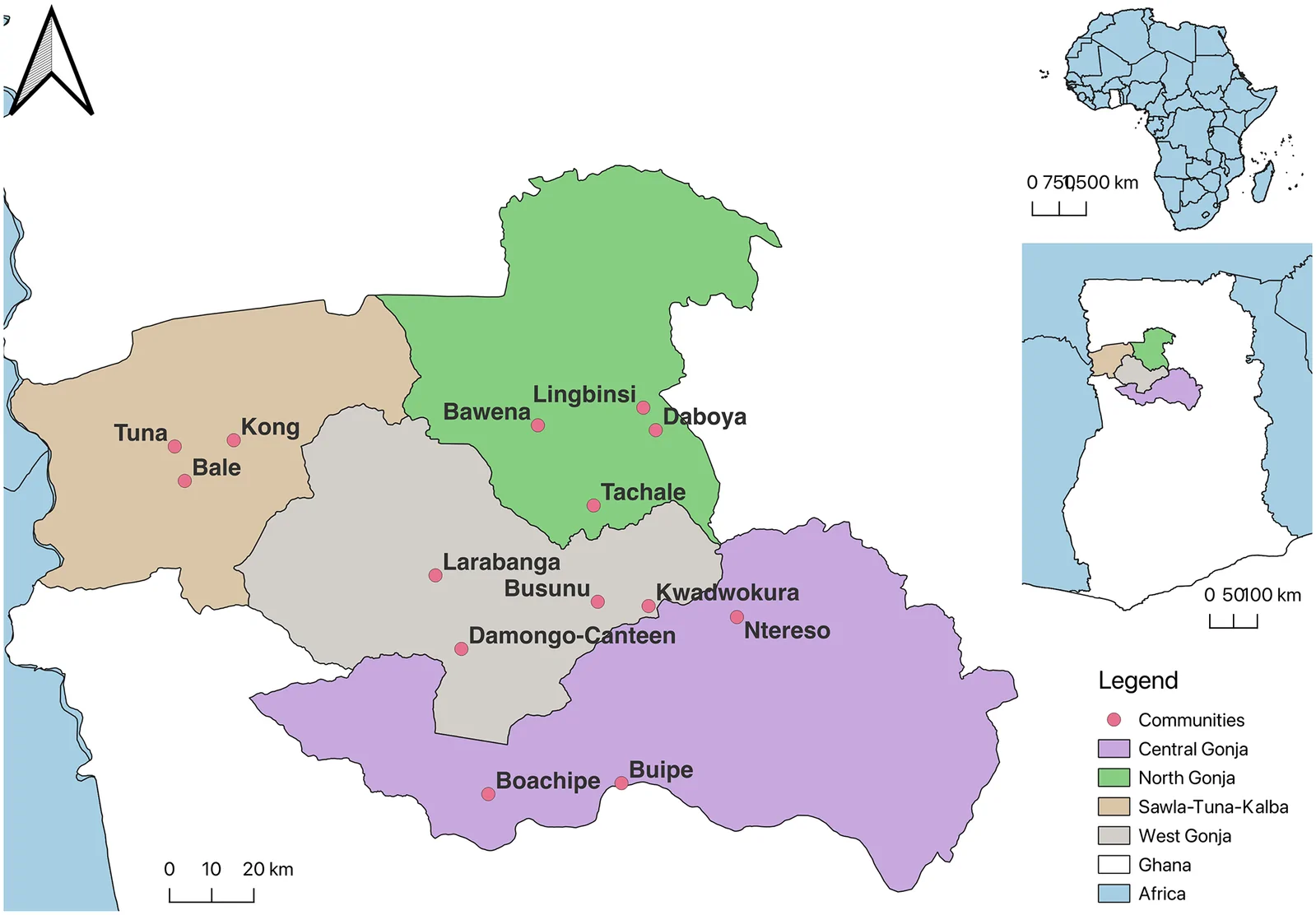
Map of study districts and communities in the Savannah Region of Ghana. The base layer for this map was accessed from the “Simplemaps – Interactive Maps & Data” website [https://simplemaps.com/gis/country/gh]. License: Creative Commons Attribution 4.0. The software for drawing this map is QGIS 3.40 [https://www.qgis.org/download/]. The terms of use / license information for the base layer image or shapefile is [https://creativecommons.org/licenses/by/4.0/].

The Savannah Region lies in the Guinea Savannah vegetation zone and it covers a total area of approximately 35,853 km^2^ making it the largest region in Ghana [37]. It lies between latitudes 8.^00^ N to 10.5^0^ N; and longitudes 0.5^0^ W to 2.5^0^ W. Mean annual rainfall and temperature are 1100 mm and 28.3 °C respectively [38]. The vegetation is mostly grasslands, shrubs and sparsely distributed clusters of drought-resistant trees [37,38]. Projections of population size and density are 625,299 and 17 per km^2^ in the year 2020 respectively [38].

### Study design

The Regional Health Directorate (RHD) and the District Health Directorates (DHD) of the Savannah Region were contacted for updates on the outbreak and obtained a list of patients that have been affected in the district (line list). The study districts and communities were then selected based on the information obtained from the line lists. Clinically, yellow fever cases are suspected based on symptoms such as fever and jaundice within 14 days of onset. These are then confirmed by laboratory diagnosis with reverse transcription PCR. This was followed by community entry / engagements to explain the study to the chiefs, elders, opinion leaders, and members of the selected communities to seek permission for surveillance. This study was a cross sectional survey involving emergency response team (ERT) moving from one outbreak community to the other. There was therefore limited time for continuous/repeated *Aedes* mosquito sampling over several days within the outbreak communities amongst other reasons. This study focused only on the affected areas of the outbreak. While the ERT moved together to conduct investigations, the Entomology Team had no opportunity to select control communities (i.e., sampling in communities with similar ecology that were unaffected by the yellow fever outbreak). Having no control community is a weakness in our investigation methods and acknowledged as a limitation of the study. The affected areas included rural, peri-urban and urban settings. Vector and viral species were identified morphologically (under cold conditions) by identification keys and molecular methods (real time reverse transcription polymerase chain reaction (real time RT-PCR)) respectively. Also, other relevant vector analyses and estimations were done to assess arbovirus transmission risk. Findings from the study were shared with the ERT and all relevant stakeholders. This informs the operational emergency response strategies of the assembled teams such as the Risk Communication and Community Engagement (RCCE), and the Vaccine and Immunization teams among others. The S6 Table shows the primers and probes used for molecular detection of arboviruses for this study.

### Selection criteria

#### Inclusion criteria.

A district was selected based on the number of recorded positive cases from the communities within that district. Between three to four communities per selected district (having reported YF cases) were chosen from the district line list after ranking them based on the reported number of YF positives and death where applicable. We acknowledge that this purposeful selection may introduce bias. However, we needed to contain the rapid spread of yellow fever by investigating such communities with all urgency.

#### Exclusion criteria.

Districts and communities having comparatively no or low numbers of YF positives (up to 2 suspected cases) or no death cases associated with YF were excluded from the study.

### Data analyses

The WHO has a threshold for estimating risk of arboviral disease transmission using the vector indices. Vector (larval and pupal) indices were estimated to assess potential risk for yellow fever outbreak in the study area using the WHO risk assessment criteria [39]. ANOVA, F-test, and T-test were used where applicable to estimate differences in variance for the *Aedes aegypti* mosquito species caught in all study districts during the entomological investigations. Multinomial logistic regression and multi-criteria analyses (MCA) were used to model local and district level risk of yellow fever transmission associated with the type of breeding sites and densities of the vector species. These model analyses were used to predict the probabilities for absence or presence of yellow fever risk based on breeding site types and vector densities.

### Entomological indices

Entomological indices (BI, CI, and HI) were used to assess risk levels. Breteaux index (BI) is defined as the number of containers found positive for larvae and/or pupae per 100 houses surveyed. This was calculated as the number of immature *Aedes*-positive containers divided by the number of houses inspected all multiplied by 100. The container index (CI) is the percentage of containers that were found positive. The CI is expressed as number of immature *Aedes*-positive water-holding containers divided by total number of inspected water-holding containers all multiplied by 100%. The house index (HI) is the percentage of houses found positive for larvae or pupae. The HI is expressed as total number of immature *Aedes*-positive houses divided by total number of inspected houses all multiplied by 100%. The respective threshold for Container Index (CI), House Index (HI), and Breteaux Index (BI) are 3.0%, 4.0%, and 5.0%. Beyond these thresholds, the risk of a given arboviral disease outbreak is deemed high. Briefly, BI, CI, and HI between 3%-20%, 4%-35%, and 5%-50% respectively is deemed sufficiently high to promote an outbreak while respective indices above 20%, 35%, and 50% are high. Pupal index (PI) or pupal productivity index (PPI) also measures the pupae per person (total number of *Aedes* pupae collected divided by the number of persons represented by the surveyed household), pupae per house (total number of *Aedes* pupae collected divided by number of houses inspected), and pupae per container depending on the surveillance goals.

### Sample size calculation

To calculate the sample size for the number of mosquitoes required for analysis, a few assumptions and considerations were made. Briefly, the population of *Aedes* mosquitoes in the study area was assumed to be infinite. This is because there are infinite populations of wildtype mosquitoes in the study area to sample. A 5% margin of error (e) was then used with a corresponding Z-score (Z) of 1.96 and a proportion of the population (p) assumed to be at least having an arboviral infection of 0.5 (i.e., 50%). The formula used for sample size calculation is as shown below.


$$\begin{array}{c} Sample\text{\textasciitilde{}size}{=Z}^{2}\text{\textasciitilde{}}*\text{\textasciitilde{}}\frac{p\left(1-p\right)}{e^{2}} \end{array}$$

The sample size estimated for analyses was at least 385 Aedes mosquitoes using the sample size formula.

### Immature and adult mosquito sampling

The mosquito surveys started on the 08/12/2021 and ended on 24/12/2021 as described below.

In the sampling of mosquitoes, each of the selected communities in the four districts was divided into 4 arbitrary quadrants. Adult and immature stages of *Aedes aegypti* mosquitoes were then sampled from each quadrant. Up to 26 houses per quadrant were inspected and sampled. Based on the WHO criteria for estimation of risk of arboviral transmission using vector indices (BI, CI, HI, and PDI), mosquito surveys were conducted in at least 100 houses (about 25 houses per quadrant) depending on the size of the community [39]. It is worth noting that instead of a maximum of 25 houses, 26 houses were surveyed in one of the arbitrary quadrant in a community due to insistent request from members of the house.

### Immature *Aedes* mosquito sampling

The larval surveys were also done using dipping and container surveys. Water storage containers (categorised into metallic, plastic, clay jar, tire, flowerpot, well, and other water holding objects) and all potential breeding sites were inspected for the larvae and pupae of *Aedes* mosquitoes. Where applicable, about 100 houses in each community were visited to inspect water storage containers for the presence of *Aedes* mosquito larvae and pupae. The number of containers inspected were recorded as either negative or positive for any of the immature stages. The number of houses inspected were also recorded. Other water storage containers in and around houses in the communities were also sampled. All immature stages (larvae and pupae) collected from different quadrant in each community were pooled. We acknowledge that pooling samples may obscure variations. These samples were then transported to a makeshift laboratory. It is worth noting that other larval species aside *Aedes* mosquitoes were sorted out. Some of the immature stages were stored in RNAlater while others were reared into adults and stored in RNAlater for about 2 weeks before analysis. The samples were then transported to the Noguchi Memorial Institute for Medical Research. The morphological species identification was done in the Entomology Laboratory while viral characterisation in mosquito samples (CHIKV, DENV, YFV, and ZIKV) was done in the Virology laboratory with an assay sensitivity of < 10 RNA copies per reaction. Sampling was done both indoors and outdoors. Higher numbers of mosquitoes were collected outdoors than indoors.

### Adult *Aedes* mosquito sampling

Three different methods were used for adult *Aedes* mosquito sampling to optimize capture efficiency for appreciable numbers. These methods included the use of BG Sentinel Traps which was set around places for *Aedes* mosquito oviposition and/or resting such as bushy areas and water storage areas in houses; Human Landing Catches (HLC) was done under the shades of trees which served as resting places for people in the households after their daily household chores or after their farmwork in the evening; and Prokopack Aspirators were used to collect adult mosquitoes in water storage containers that had adult *Aedes* mosquitoes resting in them or hovering above them. Briefly, ten (10) BG Sentinel traps (2–3 per quadrant) were set each morning in each of the selected communities from 06:00 GMT. Most communities had < 500 houses hence 10 traps were sufficient. These traps were then retrieved at 18:00 GMT on the same day of sampling in each community. HLC was done between the hours of 14:00 GMT to 18:00 GMT in each of the selected communities because *Aedes* mosquitoes are known to bite mostly at dawn and dusk. The HLC involved 3 collectors per community sampling at various points or suitable locations in the community. The collectors sat with only their legs exposed and used aspirators to catch any host-seeking insect that lands on the exposed skin. Measures were taken by these trained collectors to minimize bites. The Prokopack aspirations were used in every house visited during inspection of all water storage containers with hovering adult mosquitoes for the collection of adult *Aedes* mosquito where applicable. Sampling was done both indoors and outdoors. All adult *Aedes* mosquitoes were caught outdoors.

### Species identification and viral characterisation

#### Morphological identification of *Aedes* aegypti mosquitoes.

The morphological identification of adult mosquitoes were done using keys as described for *Aedes* [40,41] and *Culex* [42,43]. *Aedes aegypti* is a known vector in Ghana. All the two forms namely *Aedes aegypti aegypti* (cosmopolitan or domestic form) and *Aedes aegypti formosus* (sylvatic form) are found in Ghana. There are two morphological features that are used to separate *Ae. ae. aegypti* and *Ae. ae. formosus*. (i) The *Ae. ae. aegypti* has patch of pale scales on tergite 1 and a row of small white scales along posterior border of terga II-VII. (ii) The *Ae. ae. formosus* lacks a row of small white scales along posterior border of terga II-VII and median patch of scales (which are very faint even if present) on tergite 1 [40,41]. Supporting Information, S1 Text, has details of African *Aedes aegypti* identification.

#### Viral RNA extraction.

RNA was extracted from adult mosquito samples from the study sites using QIAGEN RNeasy kit according to manufacturer’s instruction [44] with a few modifications – i.e., aliquoting from 10 mosquito homogenates to make a pool. Each whole mosquito sample was put in a single 1.5 ml microtubes. About 560 µl Buffer AVL containing carrier RNA to a final concentration of 1 µl/µg for this mixture was added to the single mosquito in each microtube. About ten (10) of a 2 mm-diameter beads were added to each microtube. Each mosquito was then homogenised in this mixture for 10 seconds (an adequate time for homogenising relatively softer tissues such as mosquitoes) using the BioSpec Products Mini-Beadbeater-96 (BioSpec Products – Catalog No. NC0141170). About 56 µl of the homogenate in each microtube was aliquoted and pooled (up to ten (10) mosquito aliquots / homogenates per pool) to obtain a final volume of 560 µl homogenate per pool. RNA was then extracted following manufacturer’s instructions [44] and all pools were eluted to a total volume of 50 µl for viral detection. It is worth noting that very short homogenisation time coupled with pooling 10 whole mosquitoes per homogenate may affect assay sensitivity.

#### Detection of arboviruses.

Extracted RNA from adult *Aedes aegypti* was subjected to real time reverse transcription polymerase chain reaction (real time RT-PCR) for the detection of chikungunya, dengue, and Zika (a CDC trioplex master mix), and yellow fever (singleplex mastermix) (Supporting Information S6 Table). We opted for a singleplex yellow fever detection assay because in an ongoing outbreak, it was crucial to avoid possible target competition (where higher concentrations of other arboviral RNA will suppress amplification of YFV RNA) and also to prevent probe cross-reactivity (where YFV probe may theoretically bind to other arboviral amplicons having their targets in *flavivirus* region). Briefly, the validated CDC trioplex master mix and respective viral primers were used for the detection of chikungunya, dengue, and Zika viruses using AgPath-ID One-Step RT-PCR kit (Thermo Fisher Scientific, NY, USA) [45–47] with unique primers, probes, and controls to amplify the nSP1 region, untranscribed region, and envelope gene of the respective arboviruses (Supporting Information S6 Table). Human RNase P (internal control) was used in all assays, however, no mosquito-specific controls were used.

## Results

### Vector identification, densities, and distribution

The densities and distribution of the mosquito population caught during the yellow fever outbreak were established after their identification. The houses and the types of containers among others were recorded to estimate the number of vectors and the receptacles suitable for breeding of *Aedes* mosquito vectors. These parameters were captured under the environmental and house surveys.

A standardised environmental and house surveys were conducted across all the four districts to collect immature and adult stages of mosquitoes. The only exception was the differing number of houses survey due to varying community sizes. Total number of houses sampled in all communities selected were 1001 (minimum = 28; maximum = 102; differences in community sample size due to line list used for selection). The distribution of the houses in the four (4) districts are as follows; West Gonja 261 (26.07%); Central Gonja 197 (19.68%); North Gonja 292 (29.17%); and Sawla-Tuna-Kalba 251 (25.08%). The total number of houses positive for immature stages of *Aedes* mosquitoes were 130 (positivity rate = 12.99%). The immature stages of *Aedes* mosquitoes (645 larvae) collected after house inspections and raised in make-shift laboratory to adults for risk assessment were 57.5% (371/645), 34.7% (224/645), 4.3% (28/645), and 3.4% (22/645) for West Gonja, Central Gonja, North Gonja and Sawla-Tuna-Kalba respectively (Table 2). No adult mosquito mortality was recorded.

The breeding containers surveyed could be broadly grouped mostly under four different container types (Table 2)

The mosquito species (adult and immature stages raised to adult) sampled during the study are *Aedes aegypti aegypti*, *Aedes aegypti formosus*, and *Culex* mosquito species. Interestingly, the only *Aedes* mosquitoes caught during the study were *Aedes aegypti aegypti* and *Aedes aegypti formosus*. However, *Aedes aegypti aegypti* was not found in the Sawla-Tuna-Kalba district during sampling (Fig 2).

**Fig 2:**
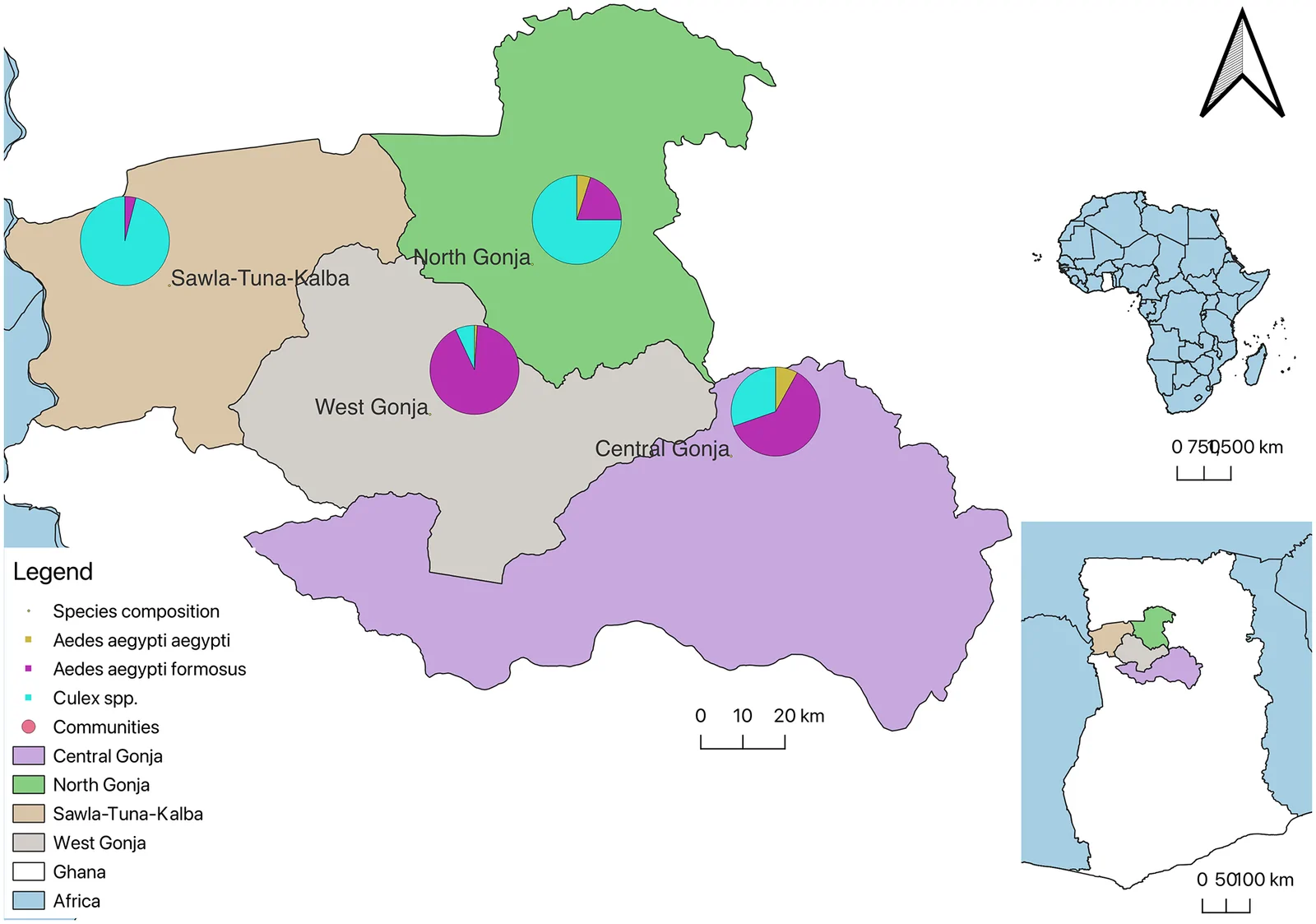
Map showing mosquitoes species caught in each district. The base layer for this map was accessed from the “Simplemaps – Interactive Maps & Data” website [https://simplemaps.com/gis/country/gh]. License: Creative Commons Attribution 4.0. The software for drawing this map is QGIS version 3.40 [https://www.qgis.org/download/]. The terms of use / license information for the base layer image or shapefile is [https://creativecommons.org/licenses/by/4.0/].

Risk of yellow fever transmission in the study communities were assess with parameters such as presence or absence of immature stage of *Aedes* mosquitoes vectors in a given breeding receptacle and the number of adult *Aedes* mosquitoes vectors caught using different adult mosquito sampling methods. This was done to estimate the risk of yellow fever infections in communities using a number of assessments detailed below.

**A**dult and immature stages of mosquitoes were collected from different breeding receptacles during the entomological investigations in the study areas (Table 2). While the highest percentages of larvae and pupae were collected from the West Gonja (62.21%, 52.53%) and Central Gonja (21.41%, 40.89%) districts, the highest numbers of breeding containers were found in North Gonja (6108 (44.40%)), and Sawla-Tuna-Kalba (6143 (44.65%)) districts. A greater number of positive breeding containers were, however, found in Central and West Gonja districts (Tables 1 and 2). In all the study districts, the study revealed that the common breeding containers were metallic and plastic (Table 2)

**Table 1:** Immature (larvae / pupae) and adult surveys in communities within the districts.

District	Community	Number of houses surveyed (%)	Number of houses positive (%, %*)	Number of larvae	Number of pupae	Number of adult *Aedes spp.* reared from larvae	Number of adult *Aedes* spp caught	Number of breeding containers surveyed (%)	Number of breeding containers positive
				(%)	(%)	(%)	(%)		(%, %*)
West Gonja	^U^Damongo - Canteen	28 (2.8)	16^a^, 2^b^ (12.3^a^, 7.1^b^, 57.1*)	1123 (28.8)	341 (28.8)	18 (2.8)	2 (7.1)	89 (0.6)	28 (16.2, 31.5*)
	^P^Busunu	101 (10.1)	17^a^, 6^b^ (13.1^a^, 21.4^b^, 16.8*)	359 (9.2)	93 (7.8)	284 (44.0)	6 (21.4)	280 (2.0)	20 (11.6, 7.1*)
	^R^Kwadwokura	35 (3.5)	2^a^ (1.5^a^, 5.7*)	15 (0.4)	4 (0.3)	15 (2.3)	0 (0.0)	93 (0.7)	2 (1.2, 2.2*)
	^P^Larabanga	97 (9.7)	18^a^, 13^b^ (13.8^a^, 46.4^b^, 18.6*)	929 (23.8)	185 (15.6)	54 (8.4)	13 (46.4)	210 (1.5)	26 (15.0, 12.4*)
Central Gonja	^R^Boachipe	35 (3.5)	6^a^ (4.6^a^, 17.1*)	26 (0.7)	57 (4.8)	6 (0.9)	0 (0.0)	183 (1.3)	8 (4.6, 4.4*)
	^P^Ntereso	61 (6.1)	4^a^, 2^b^ (3.1^a^, 7.1^b^, 6.6*)	54 (1.4)	2 (0.2)	10 (1.6)	2 (7.1)	175 (1.3)	7 (4.0, 4.0*)
	^U^Buipe	101 (10.1)	28^a^, 5^b^ (21.5^a^, 17.9^b^ 27.7*)	755 (19.4)	426 (35.9)	208 (32.2)	5 (17.9)	476 (3.5)	36 (20.8, 7.6*)
North Gonja	^P^Tachale	45 (4.5)	2^a^ (1.5^a^, 4.4*)	35 (0.9)	0 (0.0)	10 (1.6)	0 (0.0)	575 (4.2)	2 (1.2, 0.3*)
	^U^Daboya	102 (10.2)	5_a_ (3.8^a^, 4.9*)	41 (1.1)	0 (0.0)	7 (1.1)	0 (0.0)	2564 (18.6)	6 (3.5, 0.2*)
	^R^Bawena	70 (7.0)	4^a^ (3.1^a^, 5.7*)	42 (1.1)	1 (0.1)	6 (0.9)	0 (0.0)	1493 (10.9)	4 (2.3, 0.3*)
	^R^Lingbinsi	75 (7.5)	1^a^ (0.8^a^, 1.3*)	50 (1.3)	0 (0.0)	5 (0.8)	0 (0.0)	1476 (10.7)	1 (0.6, 0.1*)
Sawla-Tuna-Kalba	^R^Kong	71 (7.1)	0^a,b^ (0.0^a,b^, 0.0*)	0 (0.0)	0 (0.0)	0 (0.0)	0 (0.0)	1238 (9.0)	0 (0.0, 0.0*)
	^R^Bale	27 (2.7)	6^a^ (4.6^a^, 22.2*)	117 (3.0)	5 (0.4)	0 (0.0)	0 (0.0)	834 (6.1)	8 (4.6, 1.0*)
	^P^Tuna	100 (10.0)	19^a^ (14.6^a^, 19.0*)	288 (7.4)	16 (1.3)	18 (2.8)	0 (0.0)	2859 (20.8)	22 (12.7, 0.8*)
	^U^Sawla	53 (5.3)	2^a^ (1.5^a^, 3.8*)	66 (1.7)	56 (4.7)	4 (0.6)	0 (0.0)	1212 (8.8)	3 (1.7, 0.2*)

% = respective percentages of the total number of: houses surveyed, houses positive, larvae, pupae, adult *Aedes* spp., containers surveyed, and containers positive; * = percentages of the number positive for houses surveyed and containers positive in the respective communities; ^U^ = urban; ^P^ = peri-urban; ^R^ = rural; ^a^ = numbers and percentages (if recorded in parenthesis) of houses positive for immature stages of mosquitoes caught; and ^b^ = numbers and percentages (if recorded in parenthesis) of houses positive for adult mosquitoes caught.

**Table 2:** Types of breeding containers surveyed in the study communities.

Region	District	Community	Number of plastics containers (%)	Number of metallic containers (%)	Number of clay jars (%)	Number of tires	Number of flowerpots (%)	Number of wells	Others
						(%)		(%)	(%)
Savannah	Central Gonja	Boachipe	129 (67.5)	53 (27.7)	9 (4.7)	0 (0.0)	0 (0.0)	0 (0.0)	0 (0.0)
		Buipe	342 (66.8)	114 (22.3)	21 (4.1)	0 (0.0)	0 (0.0)	34 (6.6)	1 (0.2)
		Ntreso	91 (50.0)	55 (30.2)	36 (19.8)	1 (0.0)	0 (0.0)	0 (0.0)	0 (0.0)
	North Gonja	Bawena	681 (45.5)	662 (44.2)	70 (4.7)	84 (5.6)	0 (0.0)	0 (0.0)	0 (0.0)
		Daboya	1398 (54.4)	919 (35.8)	91 (3.5)	162 (6.3)	0 (0.0)	0 (0.0)	0 (0.0)
		Lingbinsi	471 (31.9)	862 (58.4)	98 (6.6)	46 (3.1)	0 (0.0)	0 (0.0)	0 (0.0)
		Tachale	305 (52.9)	220 (38.1)	16 (2.8)	36 (6.2)	0 (0.0)	0 (0.0)	0 (0.0)
	West Gonja	Busunu	180 (60.0)	53 (17.7)	48 (16.0)	19 (6.3)	0 (0.0)	0 (0.0)	0 (0.0)
		Canteen-Damongo	74 (63.2)	37 (31.6)	6 (5.1)	0 (0.0)	0 (0.0)	0 (0.0)	0 (0.0)
		Kwadwokura	46 (48.4)	17 (17.9)	28 (29.5)	4 (4.2)	0 (0.0)	0 (0.0)	0 (0.0)
		Larabanga	164 (69.5)	52 (22.0)	20 (8.5)	0 (0.0)	0 (0.0)	0 (0.0)	0 (0.0)
	Sawla-Tuna-Kalba	Sawla	535 (44.0)	635 (52.3)	35 (2.9)	10 (0.8)	0 (0.0)	0 (0.0)	0 (0.0)
		Tuna	1428 (49.6)	1197 (41.5)	63 (2.2)	193 (6.7)	0 (0.0)	0 (0.0)	0 (0.0)
		Bale	218 (25.9)	443 (52.6)	176 (20.9)	5 (0.6)	0 (0.0)	0 (0.0)	0 (0.0)
		Kong	467 (37.7)	705 (56.9)	37 (3.0)	29 (2.3)	0 (0.0)	0 (0.0)	0 (0.0)

The different breeding receptacles surveyed in the communities during the entomological investigations. The volumes and sizes of these receptacles though not recorded had a wide range (receptacles with size that could hold between 50 ml to about 220 litres – barrel or drum size). These water receptacles were either used for water storage or abandoned.

Only two (2) adult *Aedes aegypti* mosquitoes were caught with the BG Sentinel Trap in West Gonja District. Also, a total of 26 *Aedes aegypti* were collected using Prokopack. No mosquitoes were caught using HLC. All adult *Aedes aegypti* mosquitoes (either raised from larval collections or collected with any of the adult mosquito collection methods) were morphologically identified (Tables 3 and 4). Most of the adult *Aedes aegypti* were caught using the Prokopak aspiration method (*p*-value < 0.001) (see Supporting Information S3 Table,). However, there was no significant difference between the number of mosquitoes caught using BG-Sentinel Trap and HLC methods (*p* = 0.16) (see Tables 3 and S5 Table).

**Table 3:** Aedes aegypti mosquito sampling showing collection methods.

Region	District	Community	Stage caught / reared to adult				Place found		Total number caught
			Adult			Larva			
			B	H	P	D	Indoor	Outdoor	
Savannah	Central Gonja	Boachipe	0	0	0	10	No	Yes	10
		Buipe	0	0	5	208	No	Yes	213
		Ntreso	0	0	2	6	No	Yes	8
	North Gonja	Bawena	0	0	0	6	No	Yes	6
		Daboya	0	0	0	7	No	Yes	7
		Lingbinsi	0	0	0	5	No	Yes	5
		Tachale	0	0	0	10	No	Yes	10
	Sawla-Tuna-kalba	Sawla	0	0	0	4	No	Yes	4
		Tuna	0	0	0	18	No	Yes	18
		Bale	0	0	0	0	No	No	0
		Kong	0	0	0	0	No	No	0
	West Gonja	Busunu	2	0	4	284	No	Yes	290
		Canteen-Damongo	0	0	2	18	No	Yes	20
		Kwadwokura	0	0	0	15	No	Yes	15
		Larabanga	0	0	13	54	No	Yes	67

Table showing adult and immature sampling methods used for the entomological investigations. B = B-G Sentinel Trap; H = Human Landing Catches; P = Prokopak Aspirator; and D = Dipping (Laddles/Dippers).

**Table 4:** Aedes mosquito sampling showing adults caught and immature stages raised to adults for laboratory analyses.

District	Community	Morphological Species Identification				Total
		*Ae. aegypti aegypti*		*Ae. aegypti formosus*		
		Reared from larva to adult	Caught as adult	Reared from larva to adult	Caught as adult	
Central Gonja	Boachipe	3	0	7	0	**10**
	Buipe	18	0	190	5	**213**
	Ntreso	4	0	2	2	**8**
North Gonja	Bawena	1	0	5	0	**6**
	Daboya	0	0	7	0	**7**
	Lingbinsi	0	0	5	0	**5**
	Tachale	0	0	10	0	**10**
Sawla-Tuna-Kalba	Sawla	0	0	4	0	**4**
	Tuna	4	0	14	0	**18**
West Gonja	Busunu	3	0	281	6	**290**
	Canteen-Damongo	0	0	18	2	**20**
	Kwadwokura	0	0	15	0	**15**
	Larabanga	2	0	52	13	**67**
**Total**		**35**	**0**	**610**	**28**	**673**

Table showing larvae of *Aedes aegypti aegypti* and *Aedes aegypti formosus* raised to adults and those caught as adults for downstream analyses.

There were more *Aedes aegypti formosus* caught during the outbreak than *Aedes aegypti aegypti* (*p* < 0.001) Tables 4 and 11 (Supporting Information, S2 Table). There were significant differences between the numbers of *Ae. ae. aegypti* and *Ae. ae. formosus* caught in all study areas (*p* < 0.001) - Central Gonja and North Gonja, Central Gonja and Sawla-Tuna-Kalba, North Gonja and Sawla-Tuna-Kalba, Sawla-Tuna-Kalba and West Gonja, Central Gonja and West Gonja, as well as North Gonja and West Gonja (see S2-S4 Tables).

Pupae collected was also used to estimate risk in the study communities (Tables 5 and 6). In general, pupal index revealed high risk of yellow fever transmission in all study districts with transmission risk being low in a few communities (Daboya, Kong, Lingbinsi, and Tachale) as shown in Table 5.

**Table 5:** Pupal index estimation.

District	Community	#Persons	Pupae Caught	Pupal Index	WHO Intervention Thresholds (Low risk: pupa/person < 1; High risk: pupa/person > 1)
Central Gonja	Boachipe	8	57	7.1	High Risk
	Ntreso	3	2	0.7	Low Risk
	Buipe	52	426	8.2	High Risk
North Gonja	Tachale	0	0	NA	Low Risk
	Daboya	0	0	NA	Low Risk
	Bawena	3	1	0.3	Low Risk
	Lingbinsi	0	0	NA	Low Risk
West Gonja	Canteen-Damongo	25	341	13.6	High Risk
	Busunu	21	93	4.4	High Risk
	Kwadwokura	2	4	2.0	High Risk
	Larabanga	13	185	14.2	High Risk
Sawla-Tuna-Kalba	Kong	0	0	NA	Low Risk
	Bale	2	5	2.5	High Risk
	Sawla	5	56	11.2	High Risk
	Tuna	11	16	1.5	High Risk

Table showing pupal index estimated for the study communities using the pupae caught during entomological investigations.

**Table 6:** Breeding site or container productivity estimations (showing high risk).

District	Community	Container			Pupae counts	Average pupae per container	Breeding preference ratio (%)	Relative productivity	WHO Intervention Thresholds
		Type	Number inspected	Number positive					Low risk: container with no pupa; High risk: with pupae; High risk - priority target (container with > 10% of total pupae)
Central Gonja	Boachipe	Plastic	129	3	16	0.12	28.1	8.4	High risk
		Metallic	53	5	41	0.77	71.9	21.5	High risk - priority target
	Buipe	Plastic	342	20	130	0.38	30.5	25.4	High risk - priority target
		Metallic	114	9	72	0.63	16.9	14.1	High risk - priority target
		Clay Jar	21	7	224	10.67	52.6	43.8	High risk - priority target
	Ntreso	Clay Jar	36	4	2	0.06	100	1.1	High risk
North Gonja	Bawena	Plastic	180	4	1	0.01	100	0.3	High risk
West Gonja	Busunu	Plastic	180	7	3	0.02	3.2	1	High risk
		Metallic	53	7	74	1.4	79.6	24.7	High risk - priority target
		Clay Jar	48	6	16	0.33	17.2	5.3	High risk
	Canteen-Damongo	Plastic	74	14	268	3.62	78.6	229.1	High risk - priority target
		Metallic	37	9	48	1.3	14.1	41	High risk - priority target
		Clay Jar	6	5	25	4.17	7.3	21.4	High risk - priority target
	Kwadwokura	Clay Jar	28	1	4	0.14	100	4.2	High risk
	Larabanga	Plastic	164	15	177	1.08	95.7	75	High risk - priority target
		Clay Jar	20	5	8	0.4	4.3	3.4	High risk
Sawla-Tuna-Kalba	Bale	Clay Jar	176	6	5	0.03	NA	0.6	High risk
	Sawla	Clay Jar	35	2	56	1.6	100	4.6	High risk
	Tuna	Plastic	1428	10	12	0.01	75	0.4	High risk
		Clay Jar	63	5	4	0.06	25	0.1	High risk

Table showing productivity of the different containers inspected in the study areas.

The containers inspected in some communities (Daboya, Lingbinsi, Tachale, Bale, and Kong) were not productive (Table 6). The average pupae per container ranged between 0.00 – 10.67 while breeding preference ratio was between 0.0% - 100.0% (Table 6). Relative productivity ranged from 0 – 229.1 with the plastics in Canteen-Damongo recording the highest relative productivity. In general plastics, metals, and clay jars were very productive with their risk levels high enough to be considered priority targets in some communities including Boachipe, Buipe, Busunu, Canteen-Damongo, and Larabanga (Table 6).

In general, there was a high risk (CI > 3%, HI > 4%, and BI > 5%) of YF and other ABVs transmission in all the communities surveyed in the four districts. However, Lingbinsi and Kong communities found in North Gonja and Sawla-Tuna-Kalba districts respectively, had entomological indicies that suggested that the risk of YF and other ABVs infections in these communities were low (Table 7).

**Table 7:** Estimation of risk of yellow fever outbreak within communities in the districts.

	*Aedes aegypti* larval indices			Risk level	Common Types of breeding containers
	Container Index (CI)	House Index (HI)	Breteaux Index (BI)		
**WHO threshold (low risk)**	**<3%**	**<4%**	**<5%**		
**(outbreak / epidemic risk)**	**3-20%**	**4-35%**	**5-50%**		
**(high risk)**	**>20%**	**>35%**	**>50%**		
***West Gonja District***					
Damongo - Canteen	23.93	57.14	3.57	High risk	Plastic and metallic storage
Busunu	6.67	16.83	0.20	Outbreak / Epidemic risk	Plastic and metallic storage
Kwadwokura	2.11	5.71	0.16	Outbreak / Epidemic risk	Plastic and metallic storage
Larabanga	11.02	18.56	0.28	Outbreak / Epidemic risk	Plastic and metallic storage
***Central Gonja District***					
Boachipe	4.19	17.14	0.65	Outbreak / Epidemic risk	Plastic and metallic storage
Ntereso	3.85	6.56	0.19	Outbreak / Epidemic risk	Plastic and metallic storage
Buipe	7.03	27.72	0.36	Outbreak / Epidemic risk	Plastic and metallic storage
***North Gonja District***					
Tachale	0.35	4.44	0.10	Outbreak / Epidemic risk	Plastic and metallic storage
Daboya	0.23	4.90	0.06	Outbreak / Epidemic risk	Plastic and metallic storage
Bawena	0.27	5.71	0.08	Outbreak / Epidemic risk	Plastic and metallic storage
Lingbinsi	0.07	1.33	0.02	Low risk	Plastic and metallic storage
***Sawla-Tuna-Kalba District***					
Kong	0.00	0.00	0.00	Low risk	Plastic and metallic storage
Bale	0.95	22.22	1.10	Outbreak / Epidemic risk	Earthenware pots and metallic storage
Tuna	0.76	19.00	0.22	Outbreak / Epidemic risk	Plastic and metallic storage
Sawla	0.25	3.77	0.11	Low risk	Plastic and metallic storage

Table showing the estimated thresholds for risk analyses, conclusions for risk level, and common breeding receptacles recorded during entomological investigations.

### *Aedes aegypti* mosquito density and distribution

Yellow fever transmission risk was high (i.e., PPC > 0.5 and/or PPH > 0.5, and/or PPP > 0.5) mostly in the urban areas including Buipe, Canteen-Damongo, and Larabanga (Table 8). While percentages of containers recorded in North Gonja and Sawla-Tuna-Kalba districts was higher, percentage container productivity was higher in Central Gonja and West Gonja (Fig 3) but not significant (*p* = 0.13).

**Table 8:** Aedes aegypti mosquito pupal estimations.

District	Community	Number of persons	Containers inspected	Pupae collected	Houses surveyed	Houses infested with pupae	Pupae per container (PPC)	Pupae per house (PPH)	Pupae per person (PPP)
**Central Gonja**	**Boachipe**	86	191	57	35	4	0.2984	1.6286	0.6628
	**Buipe**	301	512	426	101	21	0.8320	4.2178	1.4153
	**Ntreso**	166	182	2	61	1	0.0110	0.0328	0.0120
**North Gonja**	**Bawena**	257	1497	1	70	1	0.0007	0.0143	0.0039
	**Daboya**	337	2570	0	102	0	0.0000	0.0000	0.0000
	**Lingbinsi**	316	1477	0	75	0	0.0000	0.0000	0.0000
	**Tachale**	148	577	0	45	0	0.0000	0.0000	0.0000
**West Gonja**	**Busunu**	306	300	93	101	6	0.3100	0.9208	0.3039
	**Canteen-Damongo**	72	117	341	28	11	2.9145	12.1786	4.7361
	**Kwadwokura**	103	95	4	35	1	0.0421	0.1143	0.0388
	**Larabanga**	233	236	185	97	3	0.7839	1.9072	0.7940
**Sawla-Tuna-Kalba**	**Bale**	74	842	0	27	1	0.0000	0.0000	0.0000
	**Kong**	303	1238	0	71	0	0.0000	0.0000	0.0000
	**Sawla**	183	1215	56	53	1	0.0461	1.0566	0.3060
	**Tuna**	369	2881	16	100	4	0.0056	0.1600	0.0434

Table 8 shows proxy for container productivity (PPC), outbreak (PPH), and transmission (PPP) risk in study districts.

**Fig 3:**
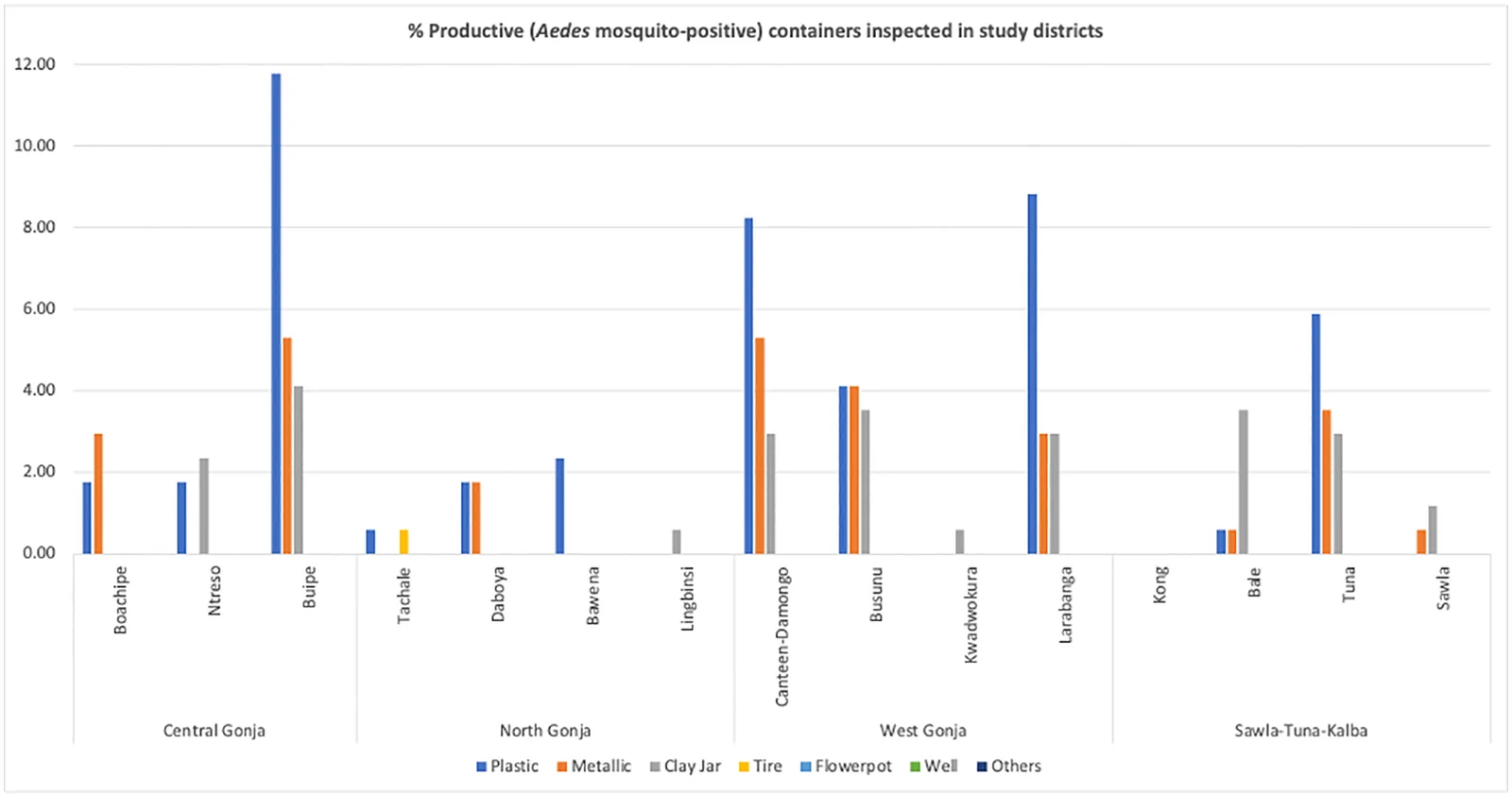
Percentage productivity of inspected containers in study districts.

### Multinomial logistic regression model and multiple criteria analysis

Container productivity was modelled to determine yellow fever virus transmission risk using multiple logistic regression and multiple criteria analysis (Table 9).

**Table 9:** Risk level modelled for study communities.

District	Community	Multiple Regression Statistic	Multinomial Regression Model	*P-*Value	Multiple Criteria Analysis		
					PPC_(weighted)_	PPC_(weighted)_ * (PPH + PPP)	Risk Level
Central Gonja	Boachipe	0.8967	Y = 0.0191 + 0.0785*P + 0.0478*M	< 0.001	0.22	0.50	Medium
	Buipe	0.7511	Y = 0.0649 + 0.0350*P + 0.0395*M + 0.0137*C	< 0.001	0.22	1.23	High
	Ntreso	NA	NA	NA	0.22	0.01	Low
North Gonja	Bawena	0.4915	Y = 0.0357 + 0.9643*P	< 0.001	0.00	0.00	Low
	Daboya	NA	NA	NA	0.00	0.00	Low
	Lingbinsi	NA	NA	NA	0.00	0.00	Low
	Tachale	NA	NA	NA	0.00	0.00	Low
West Gonja	Busunu	0.8342	Y = 0.0113 + 0.1932*P + 0.0447*M - 0.0015*C	< 0.001	0.77	0.95	Medium
	Canteen-Damongo	0.5184	Y = 0.3026 + 0.0047*P + 0.0154*M + 0.0495*C	0.046	0.77	13.06	Very High
	Kwadwokura	1	Y = 0.2500*C	< 0.001	0.77	0.12	Low
	Larabanga	0.8184	Y = 0.0069 + 0.0076*P + 0*M + 0.0016*C	< 0.001	0.77	2.09	High
Sawla-Tuna-Kalba	Bale	0.0552	Y = 0.0357 - 0.0071*C	0.99	0.01	0.00	Low
	Kong	NA	NA	NA	0.01	0.00	Low
	Sawla	1	Y = 0.0179*C	< 0.001	0.01	0.01	Low
	Tuna	0.8771	Y = 0.0065 + 0.1923*P - 0.0016*C	< 0.001	0.01	0.00	Low

Table 9 shows modelled yellow fever outbreak and transmission risk using multinomial logistic regression and multiple criteria analysis

About seven (7) categories of breeding containers (plastic, metallic, clay jar, tire, flowerpot, well, and other) were inspected for pupae. At most, only three (3) types of containers (i.e., plastic, metallic, and clay jar) were productive. No pupae were found in breeding containers from communities such as Daboya, Kong, Lingbinsi, Ntreso, and Tachale. Only one (1) container type was positive for *Aedes aegypti* mosquito pupae in Bale, Kwadwokura and Sawla (clay jars, respective Multiple R = 5.5%, 100.0%, 100.0%); and in Bawena (plastic, Multiple R = 49.2%). While Boachipe and Larabanga recorded pupae in plastic and metallic containers (respective Multiple R = 89.7% and 81.8%), in Tuna pupae were found in plastic and clay jars (Multiple R = 87.7%). The communities that had all three container types being positive for pupae were Buipe (Multiple R = 75.1%), Busunu (Multiple R = 83.4%), and Canteen-Damongo (Multiple R = 51.8%). Data from multiple criteria analysis (MCA) also revealed outbreak and transmission risk levels for yellow fever virus and other ABVs to be low in Bale, Bawena, Daboya, Kong, Kwadwokura, Lingbinsi, Ntreso, Sawla, Tachale, and Tuna. Boachipe and Busunu had Medium risk while the remaining communities had high risk. Only Canteen-Damongo was predicted to have very high risk.

### Arbovirus detection PCR

Total number of 673 adult *Aedes aegypti* mosquitoes consisting of 70 pools were screened for ABVs. Each pool had up to a maximum of 10 *Aedes aegypti* mosquito mixture making up the homogenate used for RNA extractions as describe earlier. The detection of the four arboviruses (CHIKV, DENV, YF, and ZIKV) investigated from pools of *Aedes* mosquito samples were all negative after running real time RT-PCR.

## Discussion

### Key observations

A few vital observations were made during the study. Firstly, there was adequate access to water (pipe-borne, and well) in the peri-urban (Larabanga) and urban (Buipe, Damango, Daboya, and Sawla) settings but these areas had the highest numbers of water storage containers due to unreliable pipe-borne water supply. Secondly, these peri-urban and urban dwellings had highest numbers of water storage containers infested with the immature stages of the *Aedes aegypti* mosquitoes (about 57.23% of all infested containers). Thirdly, informal interviews revealed that in the rural communities such as Lingbinsi and Kong, water is hardly stored for long periods because water was fetched from streams, rivers, and other nearby water bodies for daily house chores. Lastly, all vectors screened for the four arboviruses were negative. This may be due to only few vectors (about 4.16%) caught as adult during the outbreak. If more adult *Aedes aegypti* vectors were caught during yellow fever outbreaks there could be a high probability of getting positives after screening. An *Aedes* mosquito surveillance system may also provide ample adult mosquitoes collected within the outbreak period for risk assessment that may provide positives after testing.

Following a YF outbreak in October 2021 in the Savannah Region of Ghana, an Emergency Response Team (ERT) was assembled. This ERT was made up of experts from various institutions (including Noguchi Memorial Institute for Medical Research (NMIMR), different department under the Ghana Health Service (GHS), WASH & IPC (Water, Sanitation and Hygiene & Infection Prevention and Control), World Health Organisation (WHO) – Ghana, and Centre for Disease Prevention and Control (CDC) – Ghana) forming several groups such as Laboratory, Environmental, Vector Research, Public Health Action / Response Intervention, Case Management, WASH & IPC, Risk Communication, Community Engagement & Social Mobilisation, Logistics, and Vaccination groups among others. The ERT was tasked to assess, manage, contain and / or control the spread of the YF outbreak. This study is therefore essential because of the following reasons among others. The Savannah Region of Ghana shares porous borders with neighbouring Cote d’Ivoire and Burkina Faso. The history of arboviral disease outbreaks given in these neighbouring countries suggests that Ghana may share suitable vectors for the transmission of arboviruses with these countries. Therefore, there is the potential for the spread of YF outside of Ghana to these countries following an outbreak. Similarly, in the event of any arboviral outbreak in these neighbouring countries, there is a high potential for the spread of these arboviruses in Ghana. Despite this threat of ABV infections that may cause outbreaks in Ghana periodically, there is no established surveillance systems for ABVs in the country. This study aside solely assessing and proposing measures for containing or controlling the spread of YFV and other possible arboviruses such as CHIKV, DENV, and ZIKV using the vector indices, provides additional data which will be useful for establishing surveillance systems and planning future interventions in Ghana. Another important component of this study is the vector component. Infections leading to outbreaks of YF and other ABV diseases occur when vectors are abundant and efficient in transmitting these ABVs. It is therefore very important that surveillance is conducted during the period of an outbreak. The level of infections is usually high during epidemics. It therefore increases the chances of successfully isolating the ABVs for further studies to determine the lineages and strains of pathogens being transmitted. These are useful information for several studies including entomological research into vector competence and mosquito-virus interactions, genetic differentiation between sylvatic and urban virus lineages, host susceptibility at the molecular and immunological level [14], and vaccine studies required to treat and/or contain any future ABV outbreaks [48]. Conducting regular post-outbreak surveillance would also help avert potential ABV outbreaks or efficiently control these outbreaks whenever they occur in the future. The *Aedes aegypti* mosquito populations found in endemic areas, their vector biology, and respective densities among others would be known for the right measures and/or interventions to be put in place to effectively and efficiently handle any future outbreaks [13].

Some *Aedes* mosquitoes are known to inhabit closely to humans [49,50]. Some usually breed in water-holding containers including clay pots, metallic and plastic containers, discarded car tires, and other containers both inside and outside human houses [51,52]. Environmental and house surveys were therefore conducted for immature (larvae and pupae) [53] and adult *Aedes* mosquitoes. This was to determine distribution and density of *Aedes* mosquito vectors in the study communities. Entomological indices are recommended by the World Health Organization for monitoring vector density and evaluating control measures [39]. Pupal index (PI) or pupal productivity index (PPI) were estemated together with house index HI, container index (CI), and Breteau index (BI) which are all related to arboviral risk estimation in immature mosquito populations [39]. Pupal index were generally high in all districts. Also, metal, plastic, and clay jar containers had very high productivity in all study districts. While a few containers (< 4 litres) were found within the community serving as breeding sites, many water storage containers (ususally > 20 litres, and mostly different types of metallic, plastic, and clay jar containers) and/or *Aedes aegypti* mosquito breeding sites were found in the different houses visited. However, this observation differs from the findings of Wat’senga Tezzo et al. (2021) [54] that reported about 96.0% - 99.0% of breeding containers found outside away from houses visited. This observed contrast may be attributed to their study areas being mostly densely urban.

Our study recorded more domestic *Aedes aegypti* breeding sites and hence suggested high man-vector contact rates due to proximity of these arboviral vectors that infect humans. The major risk factors associated with the emergence of yellow fever (YF) and other arboviral diseases (including chikungunya, dengue, and Zika) were domestic water storage containers that were suitable for vector development. This observation agrees with findings from work done by Ouattara et al. (2019) [51]. Most of these water storage containers in the study district (especially outdoor metallic barrels and plastic drums) were not covered for harvesting rain [55]. Most of the covered ones atimes may have openings in the lid that may allow *Aedes aegypti* mosquitoes entry to lay eggs and contribute to vector breeding [55]. In all four (4) districts, communities (urban, peri-urban, and rural) having reliable water supply and/or potable water sources tend to have lower numbers of water storage containers. This was similar to findings from Vannavong’s and Gonzalez’s working groups [56,57]. As revealed by a Bangladesh study, reduction in the number of breeding containers correspondingly reduces vector density and hence risk of arboviral diseases [58]. Also, it was observed that communities / districts having wells as one of their sources of water had high number of water storage containers. This is also in agreement with findings from other studies that analysed water storage practices [59]. The high number of storage containers is associated with high risk of arbovirus transmission. Our observation, consistent with Gubler’s findings and worth noting, was that there were more positive breeding containers in the urban and peri-urban settings than the numbers recorded in the rural settings [60]. This is because most of these rural communities have streams or rivers from which they get water for their daily activities. Most households in the rural settings therefore may not have the need to store water for longer periods to serve as breeding sites for these *Aedes aegypti* mosquitoes. There was therefore a correspondingly lower numbers of immature stages (larvae and pupae) of *Aedes aegypti* mosquitoes obtained from breeding containers in the rural areas compared to the urban and peri-urban settlements. Considering that there were no positive containers in the rural areas may also suggest reason for the Aedine population being predominantly *Aedes aegypti formosus* which have significantly less anthropophilic behaviour thus explaining why the containers were not infested with immature *Aedes aegypti* mosquitoes.

All immature vector estimations (larval indices, pupal index, pupal productivity index, etc.) were considered for risk assessment of arbovirus transmission. When assessed with the WHO thresholds for entomological indices, this translated into high risk of ABV transmission should there be an outbreak in these study areas. This means the Savannah Region is a high risk for arbovirus transmission. Compared to the number of larvae collected for risk assessment, the number of pupae were relatively low but more reliable for immature entomological indices. Considering density and distribution of *Aedes aegypti* mosquito pupae as well as multiple criteria and regression analysis based on pupal estimations, risk of yellow fever outbreak and transmission was generally low particularly in communities of the North Gonja and Sawla-Tuna-Kalba districts [61]. However, the risk levels ranged between medium (Boachipe and Busunu) and very high (Canteen-Damongo) in the Central Gonja and West Gonja districts.

In all the communities (urban, peri-urban, and rural), the only Aedine populations obtained from the larval surveys conducted were *Aedes aegypti aegypti* and *Aedes aegypti formosus*. There were no other *Aedes* species. This observation agrees with a similar mosquito survey conducted in the northern parts of Ghana [62]. That survey found about 98% of the *Aedes* species caught to be *Aedes aegypti* which happened to be the dominant *Aedes* species and 2% being *Aedes vittatus*. An earlier work done in Damongo by another team also evaluated the community as a high risk for yellow fever transmission from vector indices evaluation (BI = 180%, CI = 44.8%, and HI = 56%) [63]. This agrees with our transmission risk assessment in Canteen-Damongo (based on our CI and HI estimations). Also, consistent with our findings, they recorded large plastic and metallic water storage drums as the dominant positive breeding receptacle [63].

Morphological identification of *Aedes aegypti* mosquitoes is highly technical and laborious and therefore not attractive to most investigators. The *Ae*. *ae*. *aegypti* population has adapted well to urban settings and responsible for most ABV transmissions in these areas while *Ae*. *ae*. *formosus* are noted for ABV transmission in forested areas and therefore important in maintaining sylvatic and intermediate YF transmissions in primates and humans respectively [14]. The predominant ABV vector found during the YF outbreak in all study districts was *Ae*. *ae*. *formosus* suggesting that they might be the *Aedes* population driving the transmission and quick spread of YF during this epidemic. This may also suggest a possible spillover of yellow fever transmission from the sylvatic transmission cycle maintained usually by non-human primates. This may have serious implications for arboviral transmission in dwellings around forest edges. After screening the vectors for yellow fever, however, they were all negative. No virus was therefore isolated from the mosquito samples for possible comparison with the strains circulating in the humans found in the Savannah Region. A couple of reasons could be attributed to this observation. Tentatively, it could be attributed to vertical transmission of arboviruses being extremely low for molecular detection given the possibility of the *Aedes aegypti* mosquitoes having reduced vectorial capacity [31]. Contrary to this observation, it has been reported that low vertical transmission is capable of maintaining disease transmission in endemic areas [64]. Again, this may be due to a possibly low infection prevalence (usually < 2.0%) in the newly emerged mosquito population [31]. It is also likely that *Aedes aegypti* mosquitoes harbouring insect-specific viruses known to subdue the virulence of arboviruses are responsible for the low vectorial capacity [65]. However, aside field surveillance, controlled infection experiments will be required to proof that a quantified increase in insect-specific viruses reduces yellow fever transmission [66]. In addition, woefully few adults *Aedes aegypti* mosquitoes were caught as adults during the outbreak. These generation of adult mosquitoes were responsible for the outbreak. However, low arbovirus prevalence in these mosquito population may lead to low possibility of arboviral detection [31].

Owing to differences in immunity of individuals and plausible herd immunity due to vaccination campaigns, the human population is usually protected against yellow fever [67]. Nonetheless, most arbovirus infections are often misdiagnosed as malaria and may therefore be underreported [68]. This highlights the importance of thorough differential diagnosis. Screening for circulating yellow fever viruses in human samples in Ghana, however, revealed isolates (West African genotype II) similar to strains identified in Côte d’Ivoire and Senegal [26]. In this outbreak, there were reports from the hospitals that the YF cases were found mostly in the nomadic populations [26] facing logistical challenges in accessing vaccination programmes thus unvaccinated due to issues with their mobility. Reports from the GHS indicated vaccination coverage within the nomads being very low. The existing national mobile vaccination strategies policy may be enhanced, for instance, to move beyond fixed-site vaccination campaigns. Emergency preparedness policy may also be improved to identify migratory routes, areas for seasonal congregation, and mechanisms that would be useful for the rapid deployment of vaccines and health personnel. These policy upgrades will guarantee successful interventions. In Ghana YF is endemic, and it is associated with severe disease in approximately 15% of cases. Notwithstanding the fact that there is high overall population immunity against YF in Ghana (88% in 2020 according to WHO-UNICEF estimates), there are pockets of the Ghanaian population that are not vaccinated. These unvaccinated individuals pose a serious threat for yellow fever transmission despite Ghana’s high vaccination coverage. Additionally, there are the unvaccinated nomadic people as well. Most of these individuals during this survey lived usually close to the forest, middle of the forest (particularly the nomadic populations), and other areas difficult to reach during vaccination campaigns. These two populations therefore remain at risk for YF (and other arboviral diseases). Aside the *Aedes aegypti* mosquito vectors, the unvaccinated individuals could be serving as resorvoirs for the maintenance / continued YF transmission. A routine YF vaccination campaign is done to vaccinate the unvaccinated populations. The contributions of the unvaccinated nomads and individual living in rural areas to arbovirus disease outbreaks has been acknowledged historically [69]. On the contrary, our study multiple criteria and regression model reveal low risk contribution to yellow fever transmission in rural communities. The current outbreak investigation found settlements of newcomer populations who had arrived after the last mass vaccination campaign and were largely unvaccinated (Source:https://www.who.int/emergencies/disease-outbreak-news/item/yellow-fever---ghana). There is therefore the potential for a rapid spread of YF within such unvaccinated populations considering that most of these vulnerable populations live in forested areas or close to the edge of forests making it easy for them to experience sylvatic arboviral spillover infections [12].

There seems to be a recent shift in transmission cycle for arbovirus outbreaks from urban to sylvatic, savannah, or intermediate [70]. Having high densities of efficient vectors such as the *Aedes aegypti* populations in the study districts, arboviral infections in the sylvatic cycle could re-emerge leading to an outbreak [12]. This study revealed a very high number of *Aedes aegypti formosus*. This is the vector speculated to maintain the savannah YF transmission cycle after picking the infection from the primates of the sylvatic (forest) transmission cycle [49,71]. In close proximity to unvaccinated people, these vectors when infective would transmit the YF virus to these individuals upon taking blood meal leading to a possible epidemic or outbreak [62]. It was therefore imperative, during the arboviral risk assessment for the YF outbreak, to ascertain the risk of arboviral infections in the study districts particularly within settlements of these vulnerable human populations using the entomological indices.

The tires found during this study were all not productive. This may be attributed to the fact that they were mostly empty or dry during the survey. In general, our arboviral risk estimation from the larval indices revealed a high risk for the transmission of YF and other arboviruses in all the study districts. However, pupal estimations from multiple regression and multiple criteria analysis in our study suggested yellow fever and ABV outbreak and transmission risk ranging from medium to very high. This may be affirmed by the presence of the arboviral vectors in the study districts, suitable vector breeding containers found in and around houses, habitual water storage practices usually in the urban communities due to unreliable supply of potable water, and water storage containers mostly uncovered and when/where covered may usually have broken lid(s) allowing access for these mosquito vectors to use them as breeding containers.

The appreciably high densities of the arboviral vector populations found in the study areas also support this risk evaluation especially in the West and Central Gonja districts where relatively large numbers of the *Aedes aegypti* vector populations were found. One of the major occupations in these study districts is farming. The vegetation is predominantly savannah. Most of these farmlands are found in and around savannah and forested areas where most of the *Aedes aegypti formosus* responsible for maintaining YF transmission in primates (monkeys and humans) usually reside in abundance breeding in rock pools, tree holes, and leaf axils among others. Farming and other human activities such as hunting, hiking, camping, and construction of new houses has brought humans close to the edge of forested lands and areas where these vectors thrive [72]. This may therefore increase man-vector contacts for those farmers having their farms in such locations.

Though in low numbers, *Aedes ae. aegypti* known to maintain the urban YF transmission was also found in every district. These two *Aedes aegypti* populations together can concurrently spread arboviral diseases at a very fast pace during an outbreak. None of the four arboviruses namely CHIKV, DENV, YFV, and ZIKV was detected after running real-time RT-PCR. During an outbreak, it is usually expected that with up to a few hundreds of these mosquitoes screened, a successful detection of any of these arboviruses could be realised. Although no arbovirus was detected molecularly with RT-PCR, data from our study suggest the two Aedine populations played vital roles in the spread of the YFV during the outbreak of YF in the Savannah Region of Ghana with the principal vector being *Aedes aegypti formosus*. There may be a few reasons for these zero detections of YF and the other arboviral diseases. Inadequate numbers of adult *Aedes aegypti* mosquitoes were caught with all the adult collection methods (Table 4). The adult mosquito collection methods used during *Aedes* mosquito sampling included human landing catches (HLC), BG Sentinel Traps, and Prokopack collections. These three adult mosquito sampling methods was therefore used to maximize the number of the adult Aedine population directly caught from the endemic areas experiencing the outbreak for arboviral detection. The adult *Aedes* mosquitoes targeted or sampled during arboviral disease outbreaks is to allow the investigators capture a sizeable proportion of the adult vector population(s) actively infecting individuals and spreading the disease during this period. However, the total adult *Aedes aegypti* mosquitoes directly caught during the YF outbreak using the three adult *Aedes* mosquito collection methods was woefully low. For instance, no adult mosquito was caught using HLC, and only 28 *Aedes aegypti* mosquitoes were caught with the other two methods (BG Sentinel Trap = 2 and Prokopack collections = 26). Gonotrophic assessment of all these adult *Aedes aegypti* mosquitoes caught revealed that they were unfed. Therefore, there was no blood meal analyses done to ascertain the possible sources of mosquito blood meal and assess the impact on or contribution to the arbovirus transmission outbreak. The other adult mosquitoes screened for YF and the other arboviruses (CHIKV, DENV, and ZIKV) were all raised from the immature stages (larvae and pupae). Secondly, the nomad population settle in forested areas away from the main communities. They, however, frequent the main communities for some supplies vital to their survival. The *Aedes aegypti formosus* population is also known to thrive and maintain YF transmission in the forested areas. The settlements of the nomads located in the forested areas place them in close proximity to these *Ae. ae. formosus* population and hence increasing nomad-vector contact. During the YF outbreak, it was realised by the Emergency Response Team that most of the patients or reported cases were nomads. These nomads are largely unvaccinated because they move around a lot from one settlement to another and therefore hardly get the opportunity to participate in vaccination programmes. They are therefore likely to served as a probable source of infections for the YF transmission / outbreak. Even if initially there were no YF infections among the nomad populations, infective *Ae. ae. formosus* mosquitoes could quickly spread YF infections to the unvaccinated nomads. In the main communities, there are pockets of individuals who have not been vaccinated against YF and can also be infected through infective bites from vectors. They can equally serve as reservoirs for YF infections to the main communities. Transmission may be sustained only in these unvaccinated individuals who are most likely to be infected. The large, vaccinated populations in the communities therefore do not allow *Aedes aegypti* mosquitoes frequently pick up YF infections when taking bloodmeal from individuals in the communities hence a higher proportion of these mosquitoes remain uninfected. Thirdly, there are insect-specific viruses known to prevent the development of arboviruses in mosquitoes. For instance, Ghana has similar vectors for transmitting arboviruses such as all the serotypes of DENV in neighbouring Burkina Faso, Côte d’Ivoire, and Togo yet DENV outbreak has never occur in Ghana before the 2021 yellow fever outbreak. Research conducted prior to the 2021 YF outbreak revealed insect-specific viruses in these vectors after molecular analysis [65]. This could possibly explain to a large extent why it is difficult detecting arboviruses in *Aedes aegypti* mosquitoes caught and screened periodically. Additionally, a reason that cannot be ignored is an individual’s strong immunity against arboviruses in endemic settings. This suppresses the ability of vectors to pick up and transmit these arboviruses from infected individuals. Arbovirus prevalence in Ghana may be underreported because they may be misdiagnosed as malaria due to some shared common symptoms at the onset of the disease. Another important reason is the issue of vectors having low vectorial capacity. With such vectors, even in the midst of circulating arboviral infections in the human population, they cannot efficiently pick up and effectively transmit these diseases. They will therefore be negative when screened for these arboviruses.

Our entomological investigations revealed breeding receptacles or breeding sites being domestic. This may have contributed to the yellow fever outbreak. There were no systems in place to routinely collect *Aedes* mosquito samples to monitor infections in these vectors. The lack of *Aedes* mosquito surveillance systems in Ghana highlights our unpreparedness towards future yellow fever and other ABVs epidemics and pandemics. With the data presented in our study, we strongly advocate for an enhanced *Aedes* mosquito surveillance systems in order to pick up early warning signs for ABV outbreaks. Indicators from our study calls for a targeted vector control public health intervention. The productive breeding receptacles were predominantly water storage containers including buckets, earthenware pots, drums, gallons, and pans (categorised as plastic, metallic, and clay jar). These containers could all store more than 10 litres of water. The indigenes in study communities must be educated on the risk and prevention of yellow fever. They should be asked to always cover these water storage containers, empty containers that had stored water for a longer period (beyond 5 days), and regularly wash them to discard immature *Aedes* mosquitoes where applicable.

## Conclusion

The yellow fever disease is endemic in Ghana hence the periodic outbreak even though about 88% of Ghanaians have been vaccinated against this virus. YFV was not detected in the mosquito samples collected from the study districts. However, the abundance of the *Aedes aegypti* populations (considereing breeding site proximity to humans) suggests that they were the vectors that probably spread the YFV during the YF outbreak. Based on the data collected during the study, *Ae. ae. formosus* would most likely be the principal vector for the transmission of arboviruses in the study districts. And the risk of arboviral disease transmission in the study districts is generally high as suggested by the immature vector indices. The number of adult *Aedes aegypti* mosquitoes caught during the entomological investigations was woefully low. Based on immature vector indices from the study, vector control / integrated vector management activities could target metal, plastic, and clay jar containers (either abandoned or for water storage) as priority breeding sites for interventions. Vector anthropophily could not be confirmed because no bloodfed adult *Aedes* mosquitoes were caught during entomological investigations thus bloodmeal analysis was not done.

## Recommendation

It will be an incentive for members of any of the study community to empty all domestic water storage containers and those abandoned in the environment (around the houses) filled with water to help reduce the breeding sources for the arboviral vectors. All storage containers filled with water must be tightly covered and emptied if these containers have no covers and/or if the water stored will not be used in a relatively short time. This is also to reduce the number of breeding sources for arboviral vectors. Indigenes living close to forest edges in these high-risk districts are also advised to wear long clothing to cover up exposed skin to prevent or minimise man-vector contacts. There is also the need for a public health policy to establish a national *Aedes* mosquito surveillance system set up for ABVs control in the interim by leveraging the existing *Anopheles* mosquito surveillance for malaria elimination and later operating as an autonomous entity in the future. This surveillance will enhance ABVs research to generate useful data for policy and decision-making relevant to plan and execute interventions during outbreaks efficiently to mitigate future outbreaks or to completely prevent outbreaks.

## Limitations

The adult *Aedes aegypti* mosquitoes collected were woefully small for any meaningful analyses. A few unfed adults were caught so analyses such as bloodmeal, vector competence, and entomological innoculation rates (EIR) among others could not be estimated. No mosquito was caught using HLC. Man-biting rates could therefore not be calculated. Consequently, a direct yellow fever risk estimation could not be established. We therefore had to use pupal estimations to predict yellow fever outbreak and transmission risk.

## Supporting information

S1 Table History of Aedes-borne arbovirus outbreaks in Africa. (PDF)

S2 Table *Aedes aegypti* aegypti and Aedes aegypti formosus used for risk assessment in all study areas. (PDF)

S3 Table Aedes aegypti aegypti and Aedes aegypti formosus reared for risk assessment in the four districts. (PDF)

S4 Table Breakdown for *Aedes aegypti aegypti* and *Aedes aegypti formosus* reared for risk assessment in the study communities. (PDF)

S5 Table Mosquitoes caught as adult for risk assessment in the study areas. (PDF)

S6 Table Primers and probes for real time reverse transcription polymerase chain reaction. (PDF)

S7 Table Breeding site or container productivity estimations (showing all estimates). (PDF)

S1 Text Important information on morphological identification of African *Aedes aegypti* mosquitoes. (PDF)
