# Supplementary material for: Entomological investigations into yellow fever outbreak in northern Ghana
Source: PLOS Glob Public Health. 2026 Oct 15;6(10):e0007346. doi: 10.1371/journal.pgph.0007346 (PMC13671538; doi:10.1371/journal.pgph.0007346)
Supplement: Supplementary file 1 [file pgph.0007346.s001.pdf]

S1 Table. History of *Aedes*-borne arbovirus outbreaks in Africa

Country	Arbovirus Transmission	Year of arbovirus epidemic / incidence	Reference
Angola	Chikungunya	1970-1971	Russo et al., 2020; Zeller et al., 2016
	Dengue	1986, 1999-2002, 2013, 2011-2018	Amarasinghe et al., 2011; Diallo et al., 2022
	Yellow fever	2016	Tomori et al., 2022; https://africacdc.org/disease/yellow-fever/
	Zika	2015	Chang et al., 2016; Marchi et al., 2020
Benin	Chikungunya		Russo et al., 2020; Zeller et al., 2016
	Dengue	1987-1993	Amarasinghe et al., 2011; Diallo et al., 2022
	Yellow fever	1996	Tomori et al., 2022
	Zika		
Brazzaville - Republic of the Congo	Chikungunya	2011, 2018	Russo et al., 2020; Zeller et al., 2016
	Dengue		
	Yellow fever		
	Zika		
Burkina Faso	Chikungunya		
	Dengue	1925, 1983-1986, 2003-2004, 2007, 2013, 2016-2017	Amarasinghe et al., 2011; Dieng et al., 2021; Im et al., 2020; Sylla et al., 2021
	Yellow fever	1996, 2008	Tomori et al., 2022; https://africacdc.org/disease/yellow-fever/
	Zika		
Cape Verde	Chikungunya		
	Dengue	2009	Amarasinghe et al., 2011; Diallo et al., 2022

	Yellow fever		
	Zika	2015	Chang et al., 2016; Marchi et al., 2020
Cameroon	Chikungunya	2006	Russo et al., 2020; Zeller et al., 2016
	Dengue	1987-1993, 1999-2003, 2006	Amarasinghe et al., 2011; Diallo et al., 2022
	Yellow fever	2009, 2010, 2012-2013, 2021-2022	Tomori et al., 2022; https://africacdc.org/disease/yellow-fever/; https://www.who.int/emergencies/disease-outbreak657 news/item/2022-DON405; https://www.who.int/emergencies/disease-outbreak-news/item/2022-DON405
	Zika		
Central African Republic (CAR)	Chikungunya	1978-1979	Russo et al., 2020; Zeller et al., 2016
	Dengue		
	Yellow fever	2008-2009, 2021-2022	Tomori et al., 2022; https://africacdc.org/disease/yellow-fever/; https://www.who.int/emergencies/disease-outbreak657 news/item/2022-DON405; https://www.who.int/emergencies/disease-outbreak-news/item/2022-DON405
	Zika		
Chad	Chikungunya		
	Dengue		

	Yellow fever	2013, 2021-2022	Tomori et al., 2022; https://africacdc.org/disease/yellow-fever/; https://www.who.int/emergencies/disease-outbreak657 news/item/2022-DON405; https://www.who.int/emergencies/disease-outbreak-news/item/2022-DON405
	Zika		
Comoros / Comores	Chikungunya	2005-2006	Russo et al., 2020; Zeller et al., 2016
	Dengue	1943-1948, 1984, 1992-1993	Amarasinghe et al., 2011; Diallo et al., 2022
	Yellow fever		
	Zika		
Cote d'Ivoire	Chikungunya		
	Dengue	1982, 1998, 1999-2002, 2008, 2016-2017	Amarasinghe et al., 2011; Dieng et al., 2021; Im et al., 2020; Sylla et al., 2021
	Yellow fever	2008, 2010-2011, 2021	Tomori et al., 2022; https://africacdc.org/disease/yellow-fever/; https://www.who.int/emergencies/disease-outbreak-news/item/2022-DON405
	Zika		
Democratic Republic of Congo (DRC)	Chikungunya	1958, 1960,1999-2000, 2003-2012, 2018	Russo et al., 2020; Zeller et al., 2016
	Dengue	1999-2001, 2007	Amarasinghe et al., 2011; Diallo et al., 2022

	Yellow fever	2010, 2012-2014, 2016, 2021-2022	Tomori et al., 2022; https://africacdc.org/disease/yellow-fever/; https://www.who.int/emergencies/disease-outbreak657 news/item/2022-DON405; https://www.who.int/emergencies/disease-outbreak-news/item/2022-DON405
	Zika		
Djibouti	Chikungunya		
	Dengue	1991, 1992	Amarasinghe et al., 2011; Diallo et al., 2022
	Yellow fever		
	Zika		
Egypt	Chikungunya		
	Dengue	1779, 1887, 1927	Amarasinghe et al., 2011; Diallo et al., 2022
	Yellow fever		
	Zika		
Eritrea	Chikungunya		
	Dengue	2005	Amarasinghe et al., 2011; Diallo et al., 2022
	Yellow fever		
	Zika		
Ethiopia	Chikungunya	2019	Russo et al., 2020; Zeller et al., 2016
	Dengue	1999-2002, 2007, 2013	Amarasinghe et al., 2011; Diallo et al., 2022
	Yellow fever	1960-1962, 2013	Tomori et al., 2022; https://africacdc.org/disease/yellow-fever/
	Zika		
Equitorial Guinea	Chikungunya	2002, 2006	Russo et al., 2020; Zeller et al., 2016

	Dengue	1999-2002	Amarasinghe et al., 2011; Diallo et al., 2022
	Yellow fever		
	Zika		
Gabon	Chikungunya	2007-2010	Russo et al., 2020; Zeller et al., 2016
	Dengue	1999-2002, 2007	Amarasinghe et al., 2011; Diallo et al., 2022
	Yellow fever	1994, 2021	Tomori et al., 2022; https://www.who.int/emergencies/disease-outbreak-news/item/2022-DON405
	Zika	2007	Chang et al., 2016; Marchi et al., 2020
Gambia	Chikungunya		
	Dengue		
	Yellow fever	1934-1935, 1978-1979	Tomori et al., 2022
	Zika	2007, 2011-2012	Chang et al., 2016; Marchi et al., 2020
Ghana	Chikungunya		
	Dengue	1932, 1987-1993, 2002-2005	Amarasinghe et al., 2011; Diallo et al., 2022
	Yellow fever	1926-1927, 1937,1996,2012,2021	Tomori et al., 2022; https://africacdc.org/disease/yellow-fever/
	Zika		
Guinea	Chikungunya		
	Dengue		
	Yellow fever	2008-2010	Tomori et al., 2022; https://africacdc.org/disease/yellow-fever/
	Zika		
Guinea Bissau	Chikungunya		
	Dengue		

	Yellow fever		
	Zika	2015	Chang et al., 2016; Marchi et al., 2020
Kenya	Chikungunya	2004, 2014-2016, 2017-2018	Russo et al., 2020; Zeller et al., 2016
	Dengue	1982, 1984-1986	Amarasinghe et al., 2011; Diallo et al., 2022
	Yellow fever	1992-1995, 2016, 2022	Tomori et al., 2022; https://africacdc.org/disease/yellow-fever/; https://www.who.int/emergencies/disease-outbreak-news/item/2022-DON405
	Zika		
La Réunion	Chikungunya	2005-2006	Russo et al., 2020; Zeller et al., 2016
	Dengue	1977-1978	Amarasinghe et al., 2011; Diallo et al., 2022
	Yellow fever		
	Zika		
Liberia	Chikungunya		
	Dengue		
	Yellow fever	1996, 2009	Tomori et al., 2022; https://africacdc.org/disease/yellow-fever/
	Zika		
Madagascar	Chikungunya	2006	Russo et al., 2020; Zeller et al., 2016
	Dengue	1943-1948, 2006	Amarasinghe et al., 2011; Diallo et al., 2022
	Yellow fever		
	Zika		
Mali	Chikungunya		
	Dengue	2008	Amarasinghe et al., 2011; Diallo et al., 2022
	Yellow fever		
	Zika	2007, 2011-2012	Chang et al., 2016; Marchi et al., 2020

Mauritania	Chikungunya		
	Dengue	2014-2020	Amarasinghe et al., 2011; Diallo et al., 2022
	Yellow fever		
	Zika		
Mauritius	Chikungunya	2005-2006	Russo et al., 2020; Zeller et al., 2016
	Dengue	2009	Amarasinghe et al., 2011; Diallo et al., 2022
	Yellow fever		
	Zika		
Mozambique	Chikungunya		
	Dengue	1964-1985, 2014-2015	Amarasinghe et al., 2011; Diallo et al., 2022
	Yellow fever		
	Zika		
Namibia	Chikungunya		
	Dengue	1999-2002, 2006	Amarasinghe et al., 2011; Diallo et al., 2022
	Yellow fever		
	Zika		
Niger	Chikungunya		
	Dengue		
	Yellow fever	2022	https://www.who.int/emergencies/disease-outbreak-news/item/2022-DON405
	Zika		
Nigeria	Chikungunya	1964, 1969, 1974	Russo et al., 2020; Zeller et al., 2016
	Dengue	1964-1968	Amarasinghe et al., 2011; Diallo et al., 2022

	Yellow fever	1925-1928, 1986-1991, 1996, 2017, 2021-2022	Tomori et al., 2022; https://africacdc.org/disease/yellow-fever/; https://www.who.int/emergencies/disease-outbreak657 news/item/2022-DON405; https://www.who.int/emergencies/disease-outbreak-news/item/2022-DON405
	Zika	1954, 1971	Chang et al., 2016; Marchi et al., 2020
Rwanda	Chikungunya		
	Dengue	1987-1993	Amarasinghe et al., 2011; Diallo et al., 2022
	Yellow fever		
	Zika		
Republic of the Congo	Chikungunya		
	Dengue		
	Yellow fever	2009, 2021-2022	Tomori et al., 2022; https://africacdc.org/disease/yellow-fever/; https://www.who.int/emergencies/disease-outbreak-news/item/2022-DON405; https://www.who.int/emergencies/disease-outbreak-news/item/2022-DON405
	Zika		
Senegal	Chikungunya	1960, 1997-1998	Russo et al., 2020; Zeller et al., 2016
	Dengue	1928, 1979, 1980-1985, 1990, 1999, 2007, 2009-2018	Amarasinghe et al., 2011; Diallo et al., 2022
	Yellow fever	1748, 1778, 1995-1996, 2010-2011	Tomori et al., 2022; https://africacdc.org/disease/yellow-fever/
	Zika	2007, 2011-2012	Chang et al., 2016; Marchi et al., 2020

Seychelles	Chikungunya	2005-2006	Russo et al., 2020; Zeller et al., 2016
	Dengue	1976-1979	Amarasinghe et al., 2011; Diallo et al., 2022
	Yellow fever		
	Zika		
Sierra Leone	Chikungunya	1978	Russo et al., 2020; Zeller et al., 2016
	Dengue		
	Yellow fever	2009, 2011	Tomori et al., 2022; https://africacdc.org/disease/yellow-fever/
	Zika	1972	Chang et al., 2016; Marchi et al., 2020
Somalia	Chikungunya	2016	Russo et al., 2020; Zeller et al., 2016
	Dengue	1982, 1985-1987, 1992-1993, 2011-2018	Amarasinghe et al., 2011; Diallo et al., 2022
	Yellow fever		
	Zika		
South Africa	Chikungunya	1956, 1975-1977	Russo et al., 2020; Zeller et al., 2016
	Dengue	1927	Amarasinghe et al., 2011; Diallo et al., 2022
	Yellow fever		
	Zika		
Sudan	Chikungunya	2005, 2018	Russo et al., 2020; Zeller et al., 2016
	Dengue	1984-1986	Amarasinghe et al., 2011; Diallo et al., 2022
	Yellow fever	1940, 2012-2013	Tomori et al., 2022; https://africacdc.org/disease/yellow-fever/
	Zika		
Tanzania	Chikungunya	1952-1953, 2007-2008	Russo et al., 2020; Zeller et al., 2016

	Dengue	1987-1993, 1999-2002, 2006, 2010, 2014	Amarasinghe et al., 2011; Diallo et al., 2022
	Yellow fever		
	Zika		
Togo	Chikungunya		
	Dengue	1987-1993, 1999-2002	Amarasinghe et al., 2011
	Yellow fever	2020	Dieng et al., 2021; Im et al., 2020; Sylla et al., 2021
	Zika		
Uganda	Chikungunya	1961-1962, 1968	Russo et al., 2020; Zeller et al., 2016
	Dengue	1999-2002	Amarasinghe et al., 2011; Diallo et al., 2022
	Yellow fever	2011, 2016, 2022	Tomori et al., 2022; https://africacdc.org/disease/yellow-fever/; https://www.who.int/emergencies/disease-outbreak-news/item/2022-DON405
	Zika	1947	Chang et al., 2016
Zambia	Chikungunya	1959	Russo et al., 2020; Zeller et al., 2016
	Dengue	1987-1993	Amarasinghe et al., 2011; Diallo et al., 2022
	Yellow fever		
	Zika		
Zanzibar	Chikungunya	1957, 1961-1962, 1971	Russo et al., 2020; Zeller et al., 2016
	Dengue	1823,1870, 2010	Amarasinghe et al., 2011; Diallo et al., 2022
	Yellow fever		
	Zika		
Zimbabwe	Chikungunya	1957, 1961-1962, 1971	Russo et al., 2020; Zeller et al., 2016
	Dengue		
	Yellow fever		
