# Supplementary material for: Entomological investigations into yellow fever outbreak in northern Ghana
Source: PLOS Glob Public Health. 2026 Oct 15;6(10):e0007346. doi: 10.1371/journal.pgph.0007346 (PMC13671538; doi:10.1371/journal.pgph.0007346)
Supplement: Supplementary file 2 [file pgph.0007346.s002.pdf]

S2 Table. *Aedes aegypti aegypti* and *Aedes aegypti formosus* used for risk assessment in all study areas

Study Area	
Ae. aegypti aegypti	*Ae. aegypti formosus*
0	0
2	4
0	0
0	3
1	3
1	32
0	5
10	41
1	18
0	17
0	8
0	13
6	37
0	1
0	4
0	5
0	7
0	2
1	1
2	1
1	0
0	2
0	1
0	2
0	2
0	1
1	2
0	1
0	1
0	5
0	10

1	74
0	11
1	69
0	12
1	94
0	19
0	2
0	2
0	1
0	4
0	1
0	1
0	1
0	1
0	6
0	1
0	10
0	5
1	16
0	8
0	7
0	9
1	12
0	0
0	0
0	0
0	0
0	0
0	2
0	2
1	2
1	3
0	1
0	2
0	1
0	2
1	1
1	0
0	1
0	1

Table 11 shows the numbers of *Aedes aegypti* populations caught in all four (4) study districts.
