# Supplementary material for: Entomological investigations into yellow fever outbreak in northern Ghana
Source: PLOS Glob Public Health. 2026 Oct 15;6(10):e0007346. doi: 10.1371/journal.pgph.0007346 (PMC13671538; doi:10.1371/journal.pgph.0007346)
Supplement: Supplementary file 3 [file pgph.0007346.s003.pdf]

S3 Table. *Aedes aegypti aegypti* and *Aedes aegypti formosus* rared for risk assessment in the four districts

Aedes aegypti aegypti and *Aedes aegypti formosus* rared for risk assessment in the four districts

Central Gonja		North Gonja		West Gonja		Sawla-Tuna-Kalba	
Ae. aegypti aegypti	*Ae. aegypti formosus*	*Ae. aegypti aegypti*	*Ae. aegypti formosus*	*Ae. aegypti aegypti*	*Ae. aegypti formosus*	*Ae. aegypti aegypti*	*Ae. aegypti formosus*
0	0	0	2	1	74	0	0
2	4	0	1	0	11	0	0
0	0	0	2	1	69	0	0
0	3	0	2	0	12	0	0
1	3	0	1	1	94	0	0
1	32	1	2	0	19	0	2
0	5	0	1	0	2	0	2
10	41	0	1	0	2	1	2
1	18	0	5	0	1	1	3
0	17	0	10	0	4	0	1
0	8			0	1	0	2
0	13			0	1	0	1
6	37			0	1	0	2
0	1			0	1	1	1
0	4			0	6	1	0
0	5			0	1	0	1
0	7			0	10	0	1
0	2			0	5		
1	1			1	16		
2	1			0	8		
1	0			0	7		
				0	9		
				1	12		

Table 12 shows the breakdown of the numbers of *Aedes aegypti* populations used for infection risk assessment in the four (4) different study districts.
