# Supplementary material for: Entomological investigations into yellow fever outbreak in northern Ghana
Source: PLOS Glob Public Health. 2026 Oct 15;6(10):e0007346. doi: 10.1371/journal.pgph.0007346 (PMC13671538; doi:10.1371/journal.pgph.0007346)
Supplement: Supplementary file 4 [file pgph.0007346.s004.pdf]

S4 Table. Breakdown for *Aedes aegypti aegypti* and *Aedes aegypti formosus* rared for risk assessment in the study communities

Central Gonja					
Boachipe		Buipe		Ntreso	
Ae. aegypti aegypti	*Ae. aegypti formosus*	*Ae. aegypti aegypti*	*Ae. aegypti formosus*	*Ae. aegypti aegypti*	*Ae. aegypti formosus*
0	0	1	32	1	1
2	4	0	5	2	1
0	0	10	41	1	0
0	3	1	18		
1	3	0	17		
		0	8		
		0	13		
		6	37		
		0	1		
		0	4		
		0	5		
		0	7		
		0	2		

North Gonja							
Bawena		Daboya		Lingbinsi		Tachale	
0	1	0	2	0	5	0	10
1	2	0	1				
0	1	0	2				
0	1	0	2				

West Gonja							
Busunu		Canteen-Damongo		Kwadwokura		Larabanga	
1	74	0	2	0	10	1	16
0	11	0	1	0	5	0	8
1	69	0	4			0	7
0	12	0	1			0	9
1	94	0	1			1	12
0	19	0	1				
0	2	0	1				
		0	6				
		0	1				

Sawla-Tuna-Kalba							
Bale		Kong		Sawla		Tuna	
0	0	0	0	0	2	1	2
0	0			0	2	1	3
0	0					0	1
0	0					0	2
						0	1
						0	2
						1	1
						1	0
						0	1
						0	1
