# Supplementary material for: Entomological investigations into yellow fever outbreak in northern Ghana
Source: PLOS Glob Public Health. 2026 Oct 15;6(10):e0007346. doi: 10.1371/journal.pgph.0007346 (PMC13671538; doi:10.1371/journal.pgph.0007346)
Supplement: Supplementary file 5 [file pgph.0007346.s005.pdf]

S5 Table. Mosquitoes caught as adult for risk assessment in the study areas

District	Commumity	B	H	P
Central Gonja	Boachipe	0	0	0
	Buipe	0	0	1
		0	0	1
		0	0	1
		0	0	1
		0	0	1
	Ntreso	0	0	1
		0	0	1
North Gonja	Bawena	0	0	0
	Daboya	0	0	0
	Lingbinsi	0	0	0
	Tachale	0	0	0
West Gonja	Busunu	1	0	1
		1	0	1
		0	0	1
		0	0	1
	Canteen-Damongo	0	0	1
		0	0	1
	Kwadwokura	0	0	0
	Larabanga	0	0	1
		0	0	1
		0	0	1
		0	0	1
		0	0	1
		0	0	1
		0	0	1
		0	0	1
		0	0	1
		0	0	1
		0	0	1
		0	0	1
		0	0	1
Sawla-Tuna-Kalba	Bale	0	0	0
	Kong	0	0	0

Sawla	0	0	0
Tuna	0	0	0

B = Biogent Sentinel Trap; H = Human Landing Catches; and P = Prokopak Aspirator Catches
