# Supplementary material for: Entomological investigations into yellow fever outbreak in northern Ghana
Source: PLOS Glob Public Health. 2026 Oct 15;6(10):e0007346. doi: 10.1371/journal.pgph.0007346 (PMC13671538; doi:10.1371/journal.pgph.0007346)
Supplement: Supplementary file 6 [file pgph.0007346.s006.pdf]

S6 Table. Primers and probes for real time reverse transcription polymerase chain reaction

Primer / Probe	Description	Sequence (5"-3")
Yellow fever virus detection (singleplex assay)		
YFS	Yellow fever sense primer	AAT AGT TGC TAG GCA ATA AAC AC
YFAS	Yellow fever antisense primer	TCC CTG AGC TTT ACG ACC AGA
YFP	Yellow fever probe	ATT CGT TCG TTG AGC GAT TAG CAG T
Other arbovirus detection (trioplex assay)		
Zika virus detection		
ZIKV-F	Zika virus sense primer	CCG CTG CCC AAC ACA AG
ZIKV-R	Zika virus antisense primer	CCA CTA ACG TTC TTT TGC AGA CAT
ZIKV-P	Zika virus probe	AGC CTA CCT TGA CAA GCA GTC AGA CAC TCA A
Dengue virus detection		
DENV-F	Dengue virus sense primer	TAG TCT RCG TGG ACC GAC AAG
DENV-R1	Dengue virus antisense primer 1	CAG TTG ACA CRC GGT TTC TC
DENV-R2	Dengue virus antisense primer 2	GGG TTG ATA CGC GGT TTC TC
DENV-P	Dengue virus probe	CGY CTW TCA ATA TGC TGA AAC GCG
Chikungunya virus detection		
CHIK-F	Chikungunya virus sense primer	ACA ATC GGT GTT CCA TCT AAA G
CHIK-R	Chikungunya virus antisense primer	GCC TGG GCT CAT CGT TAT T
CHIK-P	Chikungunya virus probe	ACA GTG GTT TCG TGT GAG GGC TAC
Human		
Rnase RP-F	Human Rnase sense primer	AGA TTT GGA CCT GCG AGC G

RP-R	Human Rnase antisense primer	GAG CGG CTG TCT CCA CAA GT
RP-P	Human Rnase probe	TTC TGA CCT GAA GGC TCT GCG CG

YFS = yellow fever sense primer, YFAS = yellow fever antisense primer, YFP = yellow fever probe; ZIKV-F = Zika virus sense primer, ZIKV-R = Zika virus antisense primer, ZIKV-P = Zika virus probe; DENV-F = dengue virus sense primer, DENV-R1 and DENV-R2 = dengue virus antisense primers, DENV-P = dengue virus probe (all DENV primers were for the detection of the four serotypes of dengue); CHIKV-F = chikungunya virus sense primer, CHIKV-R = chikungunya virus antisense primer, CHIKV-P = chikungunya virus probe; RP-F = human ribonuclease sense primer, RP-R = human ribonuclease antisense primer, and RP-P = human ribonuclease probe. All primers and probes were used in a singleplex (yellow fever) and trioplex (others) AgPath-ID One-Step RT-PCR kit (Thermo Fisher Scientific, NY, USA) assay for arbovirus detection.
