# Supplementary material for: Entomological investigations into yellow fever outbreak in northern Ghana
Source: PLOS Glob Public Health. 2026 Oct 15;6(10):e0007346. doi: 10.1371/journal.pgph.0007346 (PMC13671538; doi:10.1371/journal.pgph.0007346)
Supplement: Supplementary file 7 [file pgph.0007346.s007.pdf]

S7 Table. Breeding site or container productivity estimations (showing all estimates)

District	Community	Containers			Pupae counts	Relative productivity	WHO Intervention Thresholds
		Type	Number inspected	Number positive			Low risk: container with no pupa; High risk: with pupae; High risk - priority target (container with > 10% of total being pupae)
Central Gonja	Boachipe	Plastic	129	3	16	8.4	High risk
		Metallic	53	5	41	21.5	High risk - priority target
		Clay Jar	9	0	0	0.0	Low risk
		Tire	0	0	0	0.0	Low risk
		Flowerpot	0	0	0	0.0	Low risk
		Well	0	0	0	0.0	Low risk
		Others	0	0	0	0.0	Low risk
	Buipe	Plastic	342	20	130	25.4	High risk - priority target
		Metallic	114	9	72	14.1	High risk - priority target
		Clay Jar	21	7	224	43.8	High risk - priority target
		Tire	0	0	0	0.0	Low risk
		Flowerpot	0	0	0	0.0	Low risk
		Well	34	0	0	0.0	Low risk
		Others	1	0	0	0.0	Low risk
	Ntreso	Plastic	91	3	0	0.0	Low risk
		Metallic	55	0	0	0.0	Low risk
		Clay Jar	36	4	2	1.1	High risk
		Tire	0	0	0	0.0	Low risk

		Flowerpot	0	0	0	0.0	Low risk
		Well	0	0	0	0.0	Low risk
		Others	0	0	0	0.0	Low risk
North Gonja	Bawena	Plastic	180	4	1	0.3	Low risk
		Metallic	53	0	0	0.0	Low risk
		Clay Jar	48	0	0	0.0	Low risk
		Tire	19	0	0	0.0	Low risk
		Flowerpot	0	0	0	0.0	Low risk
		Well	0	0	0	0.0	Low risk
		Others	0	0	0	0.0	Low risk
	Daboya	Plastic	1398	3	0	0.0	Low risk
		Metallic	919	3	0	0.0	Low risk
		Clay Jar	91	0	0	0.0	Low risk
		Tire	162	0	0	0.0	Low risk
		Flowerpot	0	0	0	0.0	Low risk
		Well	0	0	0	0.0	Low risk
		Others	0	0	0	0.0	Low risk
	Lingbinsi	Plastic	471	0	0	0.0	Low risk
		Metallic	862	0	0	0.0	Low risk
		Clay Jar	98	1	0	0.0	Low risk
		Tire	46	0	0	0.0	Low risk
		Flowerpot	0	0	0	0.0	Low risk
		Well	0	0	0	0.0	Low risk
		Others	0	0	0	0.0	Low risk
	Tachale	Plastic	305	1	0	0.0	Low risk

		Metallic	220	0	0	0.0	Low risk
		Clay Jar	16	0	0	0.0	Low risk
		Tire	36	1	0	0.0	Low risk
		Flowerpot	0	0	0	0.0	Low risk
		Well	0	0	0	0.0	Low risk
		Others	0	0	0	0.0	Low risk
West Gonja	Busunu	Plastic	180	7	3	1.0	Low risk
		Metallic	53	7	74	24.7	High risk - priority target
		Clay Jar	48	6	16	5.3	High risk
		Tire	19	0	0	0.0	Low risk
		Flowerpot	0	0	0	0.0	Low risk
		Well	0	0	0	0.0	Low risk
		Others	0	0	0	0.0	Low risk
	Canteen-Damongo	Plastic	74	14	268	229.1	High risk - priority target
		Metallic	37	9	48	41.0	High risk - priority target
		Clay Jar	6	5	25	21.4	High risk - priority target
		Tire	0	0	0	0.0	Low risk
		Flowerpot	0	0	0	0.0	Low risk
		Well	0	0	0	0.0	Low risk
		Others	0	0	0	0.0	Low risk
	Kwadwokura	Plastic	46	0	0	0.0	Low risk
		Metallic	17	0	0	0.0	Low risk
		Clay Jar	28	1	4	4.2	High risk
		Tire	4	0	0	0.0	Low risk
		Flowerpot	0	0	0	0.0	Low risk

		Well	0	0	0	0.0	Low risk
		Others	0	0	0	0.0	Low risk
	Larabanga	Plastic	164	15	177	75.0	High risk - priority target
		Metallic	52	5	0	0.0	Low risk
		Clay Jar	20	5	8	3.4	High risk
		Tire	0	0	0	0.0	Low risk
		Flowerpot	0	0	0	0.0	Low risk
		Well	0	0	0	0.0	Low risk
		Others	0	0	0	0.0	Low risk
Sawla-Tuna-Kalba	Bale	Plastic	218	1	0	0.0	Low risk
		Metallic	443	1	0	0.0	Low risk
		Clay Jar	176	6	5	0.6	Low risk
		Tire	5	0	0	0.0	Low risk
		Flowerpot	0	0	0	0.0	Low risk
		Well	0	0	0	0.0	Low risk
		Others	0	0	0	0.0	Low risk
	Kong	Plastic	467	0	0	0.0	Low risk
		Metallic	705	0	0	0.0	Low risk
		Clay Jar	37	0	0	0.0	Low risk
		Tire	29	0	0	0.0	Low risk
		Flowerpot	0	0	0	0.0	Low risk
		Well	0	0	0	0.0	Low risk
		Others	0	0	0	0.0	Low risk
	Sawla	Plastic	535	0	0	0.0	Low risk

	Metallic	635	1	0	0.0	Low risk
	Clay Jar	35	2	56	4.6	High risk
	Tire	10	0	0	0.0	Low risk
	Flowerpot	0	0	0	0.0	Low risk
	Well	0	0	0	0.0	Low risk
	Others	0	0	0	0.0	Low risk
Tuna	Plastic	1428	10	12	0.4	Low risk
	Metallic	1197	6	0	0.0	Low risk
	Clay Jar	63	5	4	0.1	Low risk
	Tire	193	0	0	0.0	Low risk
	Flowerpot	0	0	0	0.0	Low risk
	Well	0	0	0	0.0	Low risk
	Others	0	0	0	0.0	Low risk

Table showing productivity of the different containers inspected in the study areas.
