# Supplementary material for: Entomological investigations into yellow fever outbreak in northern Ghana
Source: PLOS Glob Public Health. 2026 Oct 15;6(10):e0007346. doi: 10.1371/journal.pgph.0007346 (PMC13671538; doi:10.1371/journal.pgph.0007346)
Supplement: Supplementary file 8 [file pgph.0007346.s008.pdf]

S1 Text. Important information on morphological identification of African *Aedes aegypti* mosquitoes

Adult African *Aedes aegypti* morphological identification

Most working groups rely on only criteria (i) for differentiating *Aedes aegypti* subspecies neglecting criteria (ii) [1-4] It may be relatively difficult to morphologically distinguish *Ae. aegypti* populations in areas such as the Central, East, and South Africa. This is because they demonstrate phenotypic plasticity for a number of morphological traits mainly scaling patterns and colour leading to misidentification [5] hence distinguished molecularly. This observation may hold for other regional blocks in Africa including West Africa. In most African native vector mosquito populations, genetically, there are multiple divergent lineages of *Aedes aegypti* s.l. [6]. This diversity from ancestry makes the genetic breaks found between geographical locations across Africa indistinct [6]. The *Aedes aegypti* populations in most of the African regional blocks (particularly central and eastern Africa) have further subdivisions aside the classical *Aedes aegypti aegypti* (*Ae. ae. aegypti*) and *Aedes aegypti formosus* (*Ae. ae. formosus*) making it genetically difficult to distinctively identify them as *Ae. ae. aegypti* or *Ae. ae. formosus* [6]. Thus, while genetic differences are expected owing to the concept of "isolation by distance", no clear-cut or distinctive discrete clusters of *Ae. ae. aegypti* or *Ae. ae. formosus* occurs at several African regional blocks [7, 8]. There are regional blocks (including East Africa) having classic African *Ae. ae. aegypti* and *Ae. ae. formosus* populations interbreeding where their flight ranges overlap giving rise to offsprings with admixed genomes which also blurs the identification of *Ae. aegypti* populations [6]. Genetic similarity among distant *Ae. aegypti* populations in Africa have been suggested to be possibly resulting from gene flow due to human-assisted dispersal [9, 10]. Another difficulty in identification of African *Ae. aegypti* populations has been attributed to limited resolutions from traditional genetic markers such as

mitochodrial DNA [6] and microsatellites [11]. Influences from ecologically diverse habitats of African *Aedes aegypti* populations may foster local adaptation weakly impacting genetic patterns across regional blocks [7, 12]. Also, behavioural plasticity such as differences in the human biting preferences of African *Ae. aegypti* populations mars their differentiation across regional blocks [12]. Having all these genetics influencing morphology, it has been a challenge to clearly differentiate *Ae. ae. aegypti* from *Ae. ae. formosus* in most African regional blocks. However, in Ghana, these two species are morphologically distinct [3]. This is because the *Aedes aegypti* population in Ghana exhibits coexistence of morphologically distinguishable forms that do not always correspond to the genetic patterns expected elsewhere in West Africa. A study conducted in Senegal to better clarify the taxonomic status of *Aedes aegypti* populations established that the traditional morphological criterion (i.e. the absence or presence of pale scales on abdominal tergite 1) can show extensive intra-family polymorphism (i.e. families containing both *Aedes aegypti aegypti*-classified and *Aedes aegypti formosus*-classified offspring [13]. The authors therefore considered the two-subspecies classification invalid for Senegal. While Ghanaian and broader African literature recognises that clear boundaries between the traditional ecotypes are problematic, independent evidence show that *Aedes aegypti* populations occur widely and that molecular investigations have reported assignments of both *Aedes aegypti aegypti* and *Aedes aegypti formosus* in Ghana [14]. A recent study also analysed *Aedes aegypti* populations from Burkina Faso, Côte d'Ivoire, and Ghana to provide evidence of substantial genetic admixture and gene flow in these observed *Aedes aegypti* populations [15]. This suggests that Ghanaian *Aedes aegypti* populations may not have the traditional *Aedes aegypti aegypti* and *Aedes aegypti formosus* ecotypes which is worth noting. However, this conclusion was based on only few *Aedes aegypti* mosquitoes from Ho, a town in the Volta Region of Ghana. Also, while this cannot be trivialised, this genetic evaluation must be interpreted with caution because it is based on only mitochondrial DNA

assessment. Again, it has been reported that Ghanaian mosquitoes have been assigned molecularly to *Aedes aegypti aegypti* and *Aedes aegypti formosus* [16]. Captain-Esoah and colleagues' work was done in the northern parts of Ghana where our study was also conducted.

References

1. Amoa-Bosompem M, Kobayashi D, Itokawa K, Murota K, Faizah AN, Azerigyik FA, et al. Determining vector competence of *Aedes aegypti* from Ghana in transmitting dengue virus serotypes 1 and 2. Parasit Vectors. 2021;14(1):228. Epub 2021/05/01. doi: 10.1186/s13071-021-04728-z. PubMed PMID: 33926510; PubMed Central PMCID: PMCPMC8082837.

2. Futami K, Iwashita H, Higa Y, Lutiali PA, Sonye GO, Mwatele C, et al. Geographical Distribution of *Aedes aegypti aegypti* and *Aedes aegypti formosus* (Diptera: Culicidae) in Kenya and Environmental Factors Related to Their Relative Abundance. Journal of Medical Entomology. 2020;57(3):772-9. doi: 10.1093/jme/tjz233.

3. Kawada H, Higa Y, Futami K, Muranami Y, Kawashima E, Osei JHN, et al. Discovery of Point Mutations in the Voltage-Gated Sodium Channel from African Aedes aegypti Populations: Potential Phylogenetic Reasons for Gene Introgression. PLOS Neglected Tropical Diseases. 2016;10(6):e0004780. doi: 10.1371/journal.pntd.0004780.

4. Zahouli JBZ, Utzinger J, Adja MA, Müller P, Malone D, Tano Y, et al. Oviposition ecology and species composition of *Aedes* spp. and *Aedes aegypti* dynamics in variously urbanized settings in arbovirus foci in southeastern Côte d'Ivoire. Parasites & Vectors. 2016;9(1). doi: 10.1186/s13071-016-1778-9.

5. Yalwala S, Clark J, Oullo D, Ngonga D, Abuom D, Wanja E, et al. Comparative efficacy of existing surveillance tools for *Aedes aegyptiin* Western Kenya. Journal of Vector Ecology68 . 2015;40(2):301–7. doi: doi:10.1111/jvec.121.

6. Kotsakiozi P, Evans BR, Gloria-Soria A, Kamgang B, Mayanja M, Lutwama J, et al. Population structure of a vector of human diseases: *Aedes aegypti* in its ancestral range, Africa. Ecol Evol. 2018;8(16):7835-48. Epub 20180713. doi: 10.1002/ece3.4278. PubMed PMID: 30250667; PubMed Central PMCID: PMCPMC6145026.

7. Gloria-Soria A, Ayala D, Bheecarry A, Calderon-Arguedas O, Chadee DD, Chiappero M, et al. Global genetic diversity of *Aedes aegypti*. Mol Ecol. 2016;25(21):5377-95. Epub Epub 2016 Oct 14. PMID: 27671732; . doi: doi: 10.1111/mec.13866. PubMed Central PMCID: PMCPMC5123671.

8. Lee Y, Schmidt H, Collier TC, Conner WR, Hanemaaijer MJ, Slatkin M, et al. Genome-wide divergence among invasive populations of *Aedes aegypti* in California. BMC Genomics. 2019;20(1):204. Epub 20190312. doi: 10.1186/s12864-019-5586-4. PubMed PMID: 30866822; PubMed Central PMCID: PMCPMC6417271.

9. de Lourdes Munoz M, Mercado-Curiel RF, Diaz-Badillo A, Perez Ramirez G, Black WCt. Gene flow pattern among *Aedes aegypti* populations in Mexico. J Am Mosq Control Assoc. 2013;29(1):1-18. doi: 10.2987/12-6267R.1. PubMed PMID: 23687850.

10. Guagliardo SAJ, Lee Y, Pierce AA, Wong J, Chu YY, Morrison AC, et al. The genetic structure of *Aedes aegypti* populations is driven by boat traffic in the Peruvian Amazon. PLoS Negl Trop Dis. 2019;13(9):e0007552. Epub 20190918. doi: 10.1371/journal.pntd.0007552. PubMed PMID: 31532762; PubMed Central PMCID: PMCPMC6750575.

11. Dakin EE, Avise JC. Microsatellite null alleles in parentage analysis. Heredity (Edinb). 2004;93(5):504-9. doi: 10.1038/sj.hdy.6800545. PubMed PMID: 15292911.

12. Rose NH, Sylla M, Badolo A, Lutomiah J, Ayala D, Aribodor OB, et al. Climate and Urbanization Drive Mosquito Preference for Humans. Curr Biol. 2020;30(18):3570-9 e6. Epub 20200723. doi: 10.1016/j.cub.2020.06.092. PubMed PMID: 32707056; PubMed Central PMCID: PMCPMC7511451.

13. Diouf B, Dia I, Sene NM, Ndiaye EH, Diallo M, Diallo D. Morphology and taxonomic status of *Aedes aegypti* populations across Senegal. PLoS One. 2020;15(11):e0242576. Epub 20201118. doi: 10.1371/journal.pone.0242576. PubMed PMID: 33206725; PubMed Central PMCID: PMCPMC7673542.

14. Owusu-Asenso CM, Mingle JAA, Weetman D, Afrane YA. Spatiotemporal distribution and insecticide resistance status of *Aedes aegypti* in Ghana. Parasit Vectors. 2022;15(1):61. Epub 2022/02/21. doi: 10.1186/s13071-022-05179-w. PubMed PMID: 35183249; PubMed Central PMCID: PMCPMC8858493.

15. Agboli E, Zahouli JZB, Sauer FG, Sombie A, Bire YN, Adjobi CN, et al. Morphological and Genetic Heterogeneity in *Aedes aegypti* (Diptera: Culicidae) Populations Across Diverse Landscapes in West Africa. Ecol Evol. 2025;15(12):e72748. Epub 20251217. doi: 10.1002/ece3.72748. PubMed PMID: 41415319; PubMed Central PMCID: PMCPMC12710532.

16. Captain-Esoah M, Kweku Baidoo P, Frempong KK, Adabie-Gomez D, Chabi J, Obuobi D, et al. Biting Behavior and Molecular Identification of *Aedes aegypti* (Diptera: Culicidae) Subspecies in Some Selected Recent Yellow Fever Outbreak Communities in Northern Ghana. J Med Entomol. 2020;57(4):1239-45. doi: 10.1093/jme/tjaa024. PubMed PMID: 32112094.
